# Cassini Exploration of the Planet Saturn: A Comprehensive Review

**DOI:** 10.1007/s11214-020-00751-1

**Published:** 2020-10-26

**Authors:** Andrew P. Ingersoll

**Affiliations:** 1 Division of Geological and Planetary Sciences, California Institute of Technology, 1200 East California Blvd, Pasadena, CA 91125, USA

**Keywords:** Saturn, Jupiter, Cassini, Atmosphere, Interior, Giant planet

## Abstract

Before Cassini, scientists viewed Saturn’s unique features only from Earth and from three spacecraft flying by. During more than a decade orbiting the gas giant, Cassini studied the planet from its interior to the top of the atmosphere. It observed the changing seasons, provided up-close observations of Saturn’s exotic storms and jet streams, and heard Saturn’s lightning, which cannot be detected from Earth. During the Grand Finale orbits, it dove through the gap between the planet and its rings and gathered valuable data on Saturn’s interior structure and rotation. Key discoveries and events include: watching the eruption of a planet-encircling storm, which is a 20- or 30-year event, detection of gravity perturbations from winds 9000 km below the tops of the clouds, demonstration that eddies are supplying energy to the zonal jets, which are remarkably steady over the 25-year interval since the Voyager encounters, re-discovery of the north polar hexagon after 25 years, determination of elemental abundance ratios He/H, C/H, N/H, P/H, and As/H, which are clues to planet formation and evolution, characterization of the semiannual oscillation of the equatorial stratosphere, documentation of the mysteriously high temperatures of the thermosphere outside the auroral zone, and seeing the strange intermittency of lightning, which typically ceases to exist on the planet between outbursts every 1–2 years. These results and results from the Jupiter flyby are all discussed in this review.

## 1 Introduction

The planets themselves, Saturn and Jupiter, were an important scientific objective of the Cassini mission, and they are the focus of this review. The review is taken from Volume 1 of the Cassini Final Report (https://pds-rings.seti.org/cassini/). That document contains instrument-specific chapters, other discipline chapters beside the planets themselves, and a science summary with a science assessment for the complete mission. For setting priorities and planning, Cassini had five discipline working groups: Icy satellites, magnetosphere, rings, Saturn, and Titan. The report is taken from the Saturn chapter. The overlap region, where the upper atmosphere of the planet merges with the near-vacuum of space, is covered in the magnetosphere chapter if charged particles are the dominant medium, and in the Saturn chapter if neutral gases are the dominant medium

The focus is on Cassini and the discoveries it made. The report is not a complete review of giant planet atmospheres and interiors. The reference list was compiled from the Cassini instrument reports and from literature searches with keywords Cassini, Saturn OR Jupiter, atmosphere OR interior but NOT magnetosphere, rings, Titan, or other satellites. I was selective with keywords like ionosphere and aurora. I was also selective with models and laboratory work that didn’t explicitly use Cassini data. I read abstracts and tried to paraphrase the most important results, but I downloaded and read only about 20% of the papers. The final literature search was done in mid-July of 2020.

Science results are in the first 9 sections of the paper: 1. Introduction. 2. Clouds, composition and chemistry. 3. Heat flux, winds and temperatures. 4. The great storm of 2010–2011. 5. Vortices, mean flow and eddies. 6. Interior structure and rotation. 7. Upper atmosphere physics and chemistry. 8. Jupiter science results. 9. Venus atmosphere. Section 10 gives the open science questions after Cassini. Section 11 gives the Saturn portion of the scientific objectives for the prime and solstice missions. The latter was a mission extension that allowed Cassini to observe all four seasons—summer and fall in the south and winter and spring in the north. The scientific objectives are quite general, but that’s as it should be. Cassini was the first Saturn orbiter, and it was an extremely well-equipped spacecraft. It was a flagship sailing into unknown waters, and it was impossible to limit the objectives to a specific list drawn up in advance. Cassini was on a voyage of discovery, and it was wildly successful because there was much to be discovered.

## 2 Clouds, Composition and Chemistry

### 2.1 Clouds and Haze

**Fig. 1:**
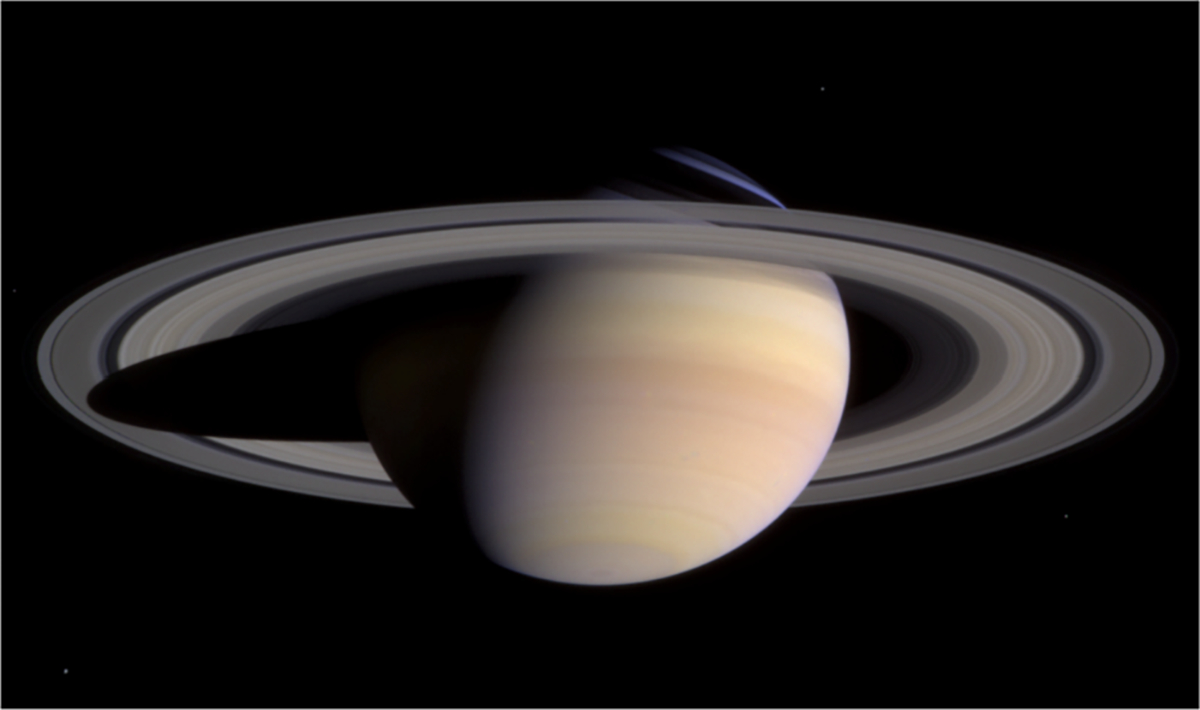
Image taken by the ISS in 2004 as Cassini was approaching Saturn. It was summer in the south, and the sunlight was driving a rich hydrocarbon chemistry. Temperatures were 10–20 K warmer in the south. The north was mostly shielded from sunlight, and the skies were blue, as shown by the sliver of atmosphere visible above the ring (PIA05389)

The temperature field, cloud properties, and composition are an interacting system that controls the energy budget of the planet and the clouds and haze that we see in the planet’s atmosphere (Fig. 1). Clouds are a tracer of the motion. They form on updrafts, which are part of the energy cycle that controls the zonal winds. Also, clouds interact with the temperature field through condensation and evaporation. Cassini, with its comprehensive array of instruments and unique perspective from different positions in the orbit, has contributed critical data on all of these objectives. In addition, Cassini has spawned exploration of Saturn from Earth-based telescopes.

Timed with the start of Cassini’s exploration of the Saturn system, Beebe (2005) presented the case for comparative study of the outer planets. Papers on the Cassini instruments and their ability to measure clouds and haze include: Baines et al. (2005) on the Visual and Infrared Mapping Spectrometer (VIMS), Porco et al. (2005) on the Imaging Science Subsystem (ISS); Flasar et al. (2004, 2005) and Jennings et al. (2017) on the Composite Infrared spectrometer (CIRS); and Janssen et al. (2013) on the Cassini RADAR radiometer. Schinder et al. (2015) give the techniques for probing the electron density and temperature structure with radio occultations.

Banfield et al. (1998) describe the use of near-infrared (IR) spectroscopy from the 5-m Hale telescope at Palomar, California to measure the altitudes and scattering properties of clouds and haze. Similarly, Sanchez-Lavega et al. (2004) described Hubble Space Telescope observations of cloud morphology in Saturn’s atmosphere. Temma et al. (2005) used Earth-based telescopic observations to probe the vertical structure of Saturn’s cloud layers. Using Hubble Space Telescope (HST) data, Perez-Hoyos et al. (2006) documented short-term variability (weeks to months) associated with cloud reflectivity of the belts and zones. West et al. (2009) gives a comprehensive survey of clouds and haze in Saturn’s atmosphere as inferred from Cassini VIMS and ISS, as well as HST and other Earth-based telescopes. Generally cloud particles form by condensation of simple molecules, and haze particles form by condensation of complex molecules that are produced by photochemistry. Ingersoll et al. (2018) present images taken during the last few months of the mission at resolutions down to 0.5 km per pixel. The interesting features include filamentary clouds coherent over 20,000 km and puffy clouds resembling terrestrial cumulus. Spilker (2019) gives the highlights and discoveries obtained during Cassini’s final year, including the some of the highest-resolution information about the interior and atmosphere.

Fletcher et al. (2007b) used Cassini CIRS data to probe the vertical and horizontal temperature structure and its relation to the belt and zone cloud structure. Roman et al. (2013) used ISS data to probe the layered cloud structure from the haze layers at 40 mbar to tropospheric clouds at 2.5 bar. Laraia et al. (2013) used Cassini RADAR radiometer data to probe the vertical distribution of NH_3_ to depths below its nominal cloud base. Barstow et al. (2016) use Cassini VIMS to document a photochemical origin for the stratospheric haze and either NH_3_ or NH_4_SH for the deeper tropospheric cloud. Using Cassini ISS data, Dyudina et al. (2016) and Perez-Hoyos et al. (2016) quantified the way the atmosphere scatters light, including the phase function and the spherical and Bond albedos.

Del Genio et al. (2009) give a comprehensive review of Saturn’s atmospheric structure and dynamics. The early data come from the Voyager flybys and Earth-based telescopes and then from the remote-sensing instruments on Cassini. The review covers the deep atmosphere, the troposphere, the stratosphere, and dynamical features such as the jets, waves, discrete features. Del Genio and Barbara (2016) provide a classification scheme based on cloud morphology for revealing the underlying dynamics.

### 2.2 Elemental and Isotopic Abundances

Counted by the number of atoms, the Sun is approximately 99.9% hydrogen/helium and 0.1% everything else. In our solar system, Jupiter comes closest to having the same abundance ratios relative to H as the Sun and Saturn comes next, with higher heavy-element ratios than Jupiter. Abundance ratios relative to H that are different from the solar ratio provide observational constraints for the formation and evolution of the planet.

The helium to hydrogen ratio in the atmosphere reflects the value in the solar nebula when the planets were forming as well as the degree to which helium has settled toward the core. Settling is thought to happen because helium becomes immiscible in metallic hydrogen, which is the dominant constituent at megabar pressures. The settling releases gravitational potential energy, which affects the cooling history and planetary evolution. In principle, helium is detected by remote sensing through its effect on the molecular weight of the gas. One uses occultation data to get density vs. altitude, and from the hydrostatic equation one gets density vs. pressure. Then one uses infrared data to get temperature vs. pressure, and with the equation of state one can solve for the molecular weight. The method was used for Jupiter and Saturn using Voyager data (Conrath et al. 1984), but the Galileo probe gave a different result for Jupiter and forced a reanalysis for Saturn (Conrath and Gautier 2000). Cassini has collected excellent data using CIRS for the temperature and occultations by several instruments—radio science, VIMS, and the Ultraviolet Imaging Spectrograph (UVIS), but getting a consistent estimate of the helium abundance is difficult. The occultation result depends on the ray path, and that depends on unknown winds and temperatures above the occultation point. The most recent estimate (Koskinen and Guerlet 2018) uses Cassini UVIS for the occultations and CIRS for temperature versus pressure. They get a helium mass fraction Y of 0.16−0.22, which is about 70% of the protosolar mass fraction of 0.27, implying that a certain amount of settling has occurred in Saturn’s interior.

Before Cassini reached Saturn, the C/H abundance ratio was known to be higher on Saturn than on Jupiter, which was known to be higher than that on the Sun (Atreya et al. 1999, 2003; Owen and Encrenaz 2003). The C/H ratio is perhaps the most accurately known ratio of all the heavy elements because its molecular form, methane, doesn’t condense at Saturn temperatures and isn’t readily destroyed in chemical reactions. Cassini CIRS probes the far-infrared rotational lines of the molecule and has determined a volume mixing ratio of (4.7 ± 0.2) × 10^−3^, corresponding to a C/H enrichment relative to solar of 10.4 (Fletcher et al. 2009b). This is more than a factor of 2 greater than that for Jupiter but consistent with conventional models of the formation and evolution of the giant planets, which predict increasing ratios to H of the heavy element abundances relative to the Sun from Jupiter to Neptune (Atreya et al. 2019).

Saturn’s greater enrichment is also qualitatively consistent with the masses and radii of the two giant planets. These, coupled with the gravitational moments, rotation rate, and an equation of state, give information about the core mass and amount of heavy elements in the molecular envelope (Guillot 1999; Nettelmann et al. 2013). Chemical models preceding the arrival of Galileo and Cassini listed many gases that were potentially observable in the atmospheres of Jupiter and Saturn (Fegley and Lodders 1994). Water is crucial because O is the third most abundant element after H and He, but the vapor pressure is so low at the tops of the clouds that Cassini could not detect it. Methane is much more volatile than water, so it does not condense in Saturn’s atmosphere. Thus the C/H ratio derived from methane is likely to be representative of the planet as a whole.

The high C/H ratio is taken as evidence of heavy element enrichment in general and has implications for possible differential sedimentation in the planet’s interior over its lifetime (Mousis et al. 2006). Measurements of NH_3_, PH_3_, C_2_H_2_, C_2_H_6_, and CH_4_ all are consistent with these gases trapped as clathrate hydrate in the feeding zone of Saturn as the planets were forming (Hersant et al. 2008). Clathrate hydrates require water, and our lack of knowledge of the oxygen abundance allows for a number of interior models with a range of values for the C/O ratio (Lunine 2011). CIRS measurements suggest that Saturn’s D/H ratio is less than that for Jupiter, which mostly reminds us that our understanding planetary formation and evolution is incomplete (Pierel et al. 2017).

Although ammonia is the main carrier of nitrogen on Jupiter and Saturn, the isotopic ratio ^15^N/^14^N can reveal insights into the molecular carrier (N_2_ versus NH_3_) in the protoplanetary nebula. The Saturnian and Jovian isotopic ratios appear indistinguishable (Abbas et al. 2004; Fletcher et al. 2014; Fouchet et al. 2004a) and distinctly lower in ^15^N than Titan. That result favors accretion of primordial N_2_ on both planets, either in the gas phase from the solar nebula or as ices formed at very low temperature (Fletcher et al. 2014), and a different source reservoir for the ^15^N-enriched atmosphere of Titan.

### 2.3 Variability of Trace Gases

The gaseous composition of a giant planet atmosphere varies due to several processes: condensation and precipitation that remove the particles from the gas, updrafts and downdrafts that alter the chemical equilibrium of the various reacting species, and photochemical reactions that lead to dissociation and/or ionization. In addition, the upper atmosphere may receive molecules and solid particles from the rings and satellites.

**Fig. 2:**
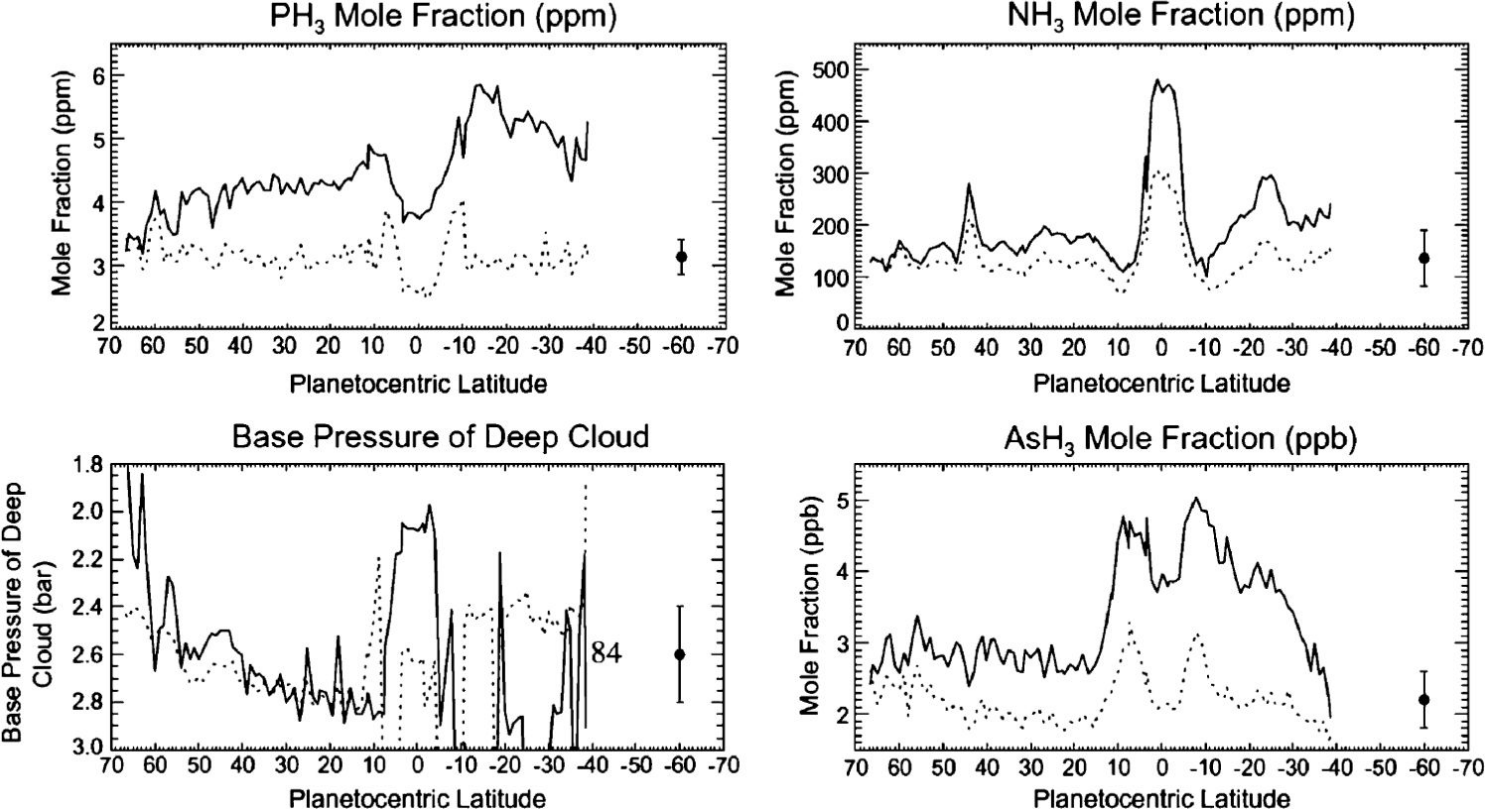
Tropospheric chemical abundances from Cassini VIMS. On first look, the profiles do not tell a consistent story. NH_3_ shows a high mole fraction at the equator, consistent with upwelling there and downwelling on either side at 10–12° latitude, but PH_3_ and AsH_3_ show low abundances at the equator, consistent with downwelling. The data may be indicating a double Hadley cell, with rising motion at the higher altitudes at the equator and sinking motion deeper down (Fletcher et al. 2011a)

Fletcher et al. (2011a) review the elements that appear in simple compounds that are detectable in VIMS spectra, including PH_3_, NH_3_, AsH_3_, GeH_3_, CO, CH_3_D, and ^13^CH_4_. They use VIMS 4.6–5.1 μm nightside spectra and solve for cloud properties and molecular abundances in the 1–4 bar pressure range. Figure 2 is the result, and there seem to be contradictions in whether there is upwelling or downwelling at the equator. NH_3_ is removed from the gas by condensation and precipitation, but only above the 1.5 bar level. PH_3_ and AsH_3_ are removed from the gas by chemical reactions at pressures ≤ 3 bars. The VIMS is seeing variability at different altitudes. Fletcher et al. calculate that the maximum of NH_3_ at the equator indicates equatorial upwelling at P < 1.3 bars, and the minima of PH_3_ and AsH_3_ at the equator indicate equatorial downwelling at P > 2.8 bars. In other words, Fletcher et al. are postulating two stacked, oppositely-directed Hadley cells based on their data. Clouds make the inversion non-unique, and the authors concede that the idea is speculative. The 2-cell idea originated with the Galileo mission to Jupiter (Ingersoll et al. 2000; Gierasch et al. 2000; Showman and de Pater 2005) to explain the preponderance of lightning in the (cyclonic) belts despite strong evidence of upwelling in the upper troposphere of the (anticyclonic) zones (Gierasch et al. 1986).

Fouchet et al. (2009) give a comprehensive review of Saturn composition and chemistry. Howett et al. (2007) and Guerlet et al. (2009) focus on C_2_H_2_, C_2_H_6_, and C_3_H_8_, which are produced above the 1 mbar level by photodissociation of methane. They use Cassini CIRS data to infer a stratospheric circulation from south to north during the southern summer. Guerlet et al. (2010) added C_3_H_8_ and CH_3_C_2_H to the list of tracers and again found evidence of upwelling in the south and downwelling in the north. Fletcher et al. (2007a) also used CIRS data, focusing on phosphine, PH_3_. It is in chemical equilibrium below the cloud tops and is destroyed by photolysis in the stratosphere. The altitudes of sources and sinks are opposite to that of C_2_H_2_ and C_2_H_6_, but the inferred circulation is similar.

Cassini CIRS data stimulated photochemical modeling in order to quantify the inferred circulations (Dobrijevic et al. 2011; Hue et al. 2015). The data also stimulated laboratory measurements (Auwera et al. 2007; Devi et al. 2014) to refine the infrared absorptions of these gases. Gases like CH_4_, C_2_H_2_, and C_2_H_6_ absorb and emit in the infrared, and their spatial and temporal variations create temperature variations, which in turn affect the circulation. These interactions are studied with a 2-D photochemical model (Hue et al. 2016). They obtain “moderate” agreement with the observed species distributions, but they say that more sophisticated 2D and 3D representations of transport are needed.

Intensified Earth-based observations are a further desirable result of Cassini’s mission to Saturn. Some of the observations immediately preceded the outpouring of Cassini data (Kim and Geballe 2005; Tejfel 2005), both to extend the range of temporal variability and to compare inferences based on different data sets. For instance, microwaves are sensitive to ammonia vapor at deeper levels than those probed by infrared radiation. The Cassini RADAR used in passive mode collects thermal radiation at 2.2 cm wavelength. With laboratory measurements of the ammonia opacity (Hanley et al. 2009), the Cassini 2.2 cm data were used to infer depletion of ammonia below the expected base of the ammonia cloud (Janssen et al. 2013; Laraia et al. 2013). This depletion is observed on Jupiter as well and has not been fully explained. The NH_3_ abundance is controlled mostly by condensation and evaporation, which is more tied to the cloud structure, so it reveals different aspects of the circulation (Hurley et al. 2012b) than that of the hydrocarbons.

In giant planet atmospheres, the principal oxygen-bearing molecules are H_2_O and CO, with the latter in equilibrium at higher temperatures and pressures than the former. However, water is frozen out at high altitudes, so the presence of CO_2_ at the 1 mbar level suggests a source outside the planet. Abbas et al. (2013) argued that the oxygen source is interstellar dust. Cavalie et al. (2019) argued that the plumes of Enceladus are the most likely source. Abbas et al. use Cassini CIRS data and Cavalie et al. use Hershel data, but the latter group base their argument on the fact that stratospheric water peaks at the equator whereas interstellar dust would not.

## 3 Heat Flux, Winds and Temperatures

### 3.1 Radiation Budget

**Fig. 3:**
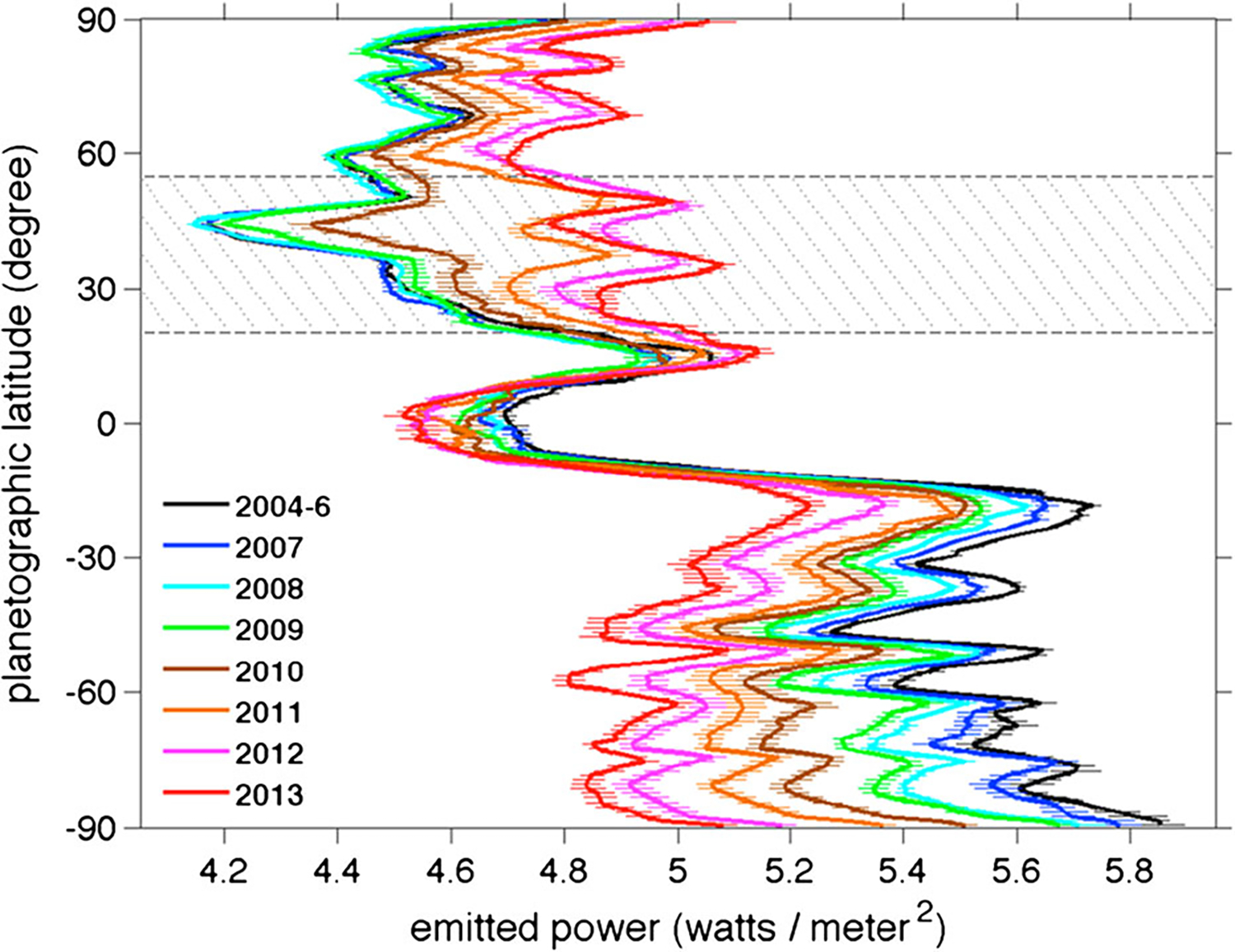
Thermal emission of the planet in units of W m^−2^ from Cassini CIRS. In the south the season was early summer in 2004 and mid fall in 2013. The decrease in thermal emission in the south was due to cooling of the atmosphere. Comparing the years before and after 2011, it is clear that the great storm increased the thermal emission by 5–10% in the 30–40° latitude band, and that the anomaly persisted for several years (Li et al. 2015)

The internal heat coming out of the planet is a fundamental quantity relevant to formation and evolution. Because giant planets in our solar system are massive and their surfaces are cold, they cool off slowly. Their interiors are still warm, and they are still releasing some of their heat of formation. To measure the internal power one takes the difference between the emitted infrared power and the absorbed solar power, which is the difference between the incident and reflected sunlight. Using Voyager data, Hanel et al. (1983) estimated the effective emission temperature as 95.0 ± 0.4 K, corresponding to an average emitted heat flux of 4.62 ± 0.8 Wm^−2^, and the ratio of emitted power to absorbed power as 1.78 ± 0.09. Thus the internal power divided by the absorbed solar power is 0.78. Using data from Cassini CIRS, Li et al. (2010) estimated the emitted heat flux as 4.952±0.035 Wm^−2^ during 2004–2009, although it decreased by 2% during that time and was 16% higher in the southern hemisphere than in the northern hemisphere. These were likely seasonal changes associated with the approach of vernal equinox in 2009. Li et al. (2015) measured the emitted power vs. latitude from 2004 to 2013 and documented the 2010 great storm’s effect, which was to increase the global emitted power by 2% and the power at the latitude of the storm by 9% (Fig. 3). These changes were in addition to a seasonal warming of the north and cooling of the south from 2004 to 2013.

### 3.2 Seasonal Variations

**Fig. 4:**
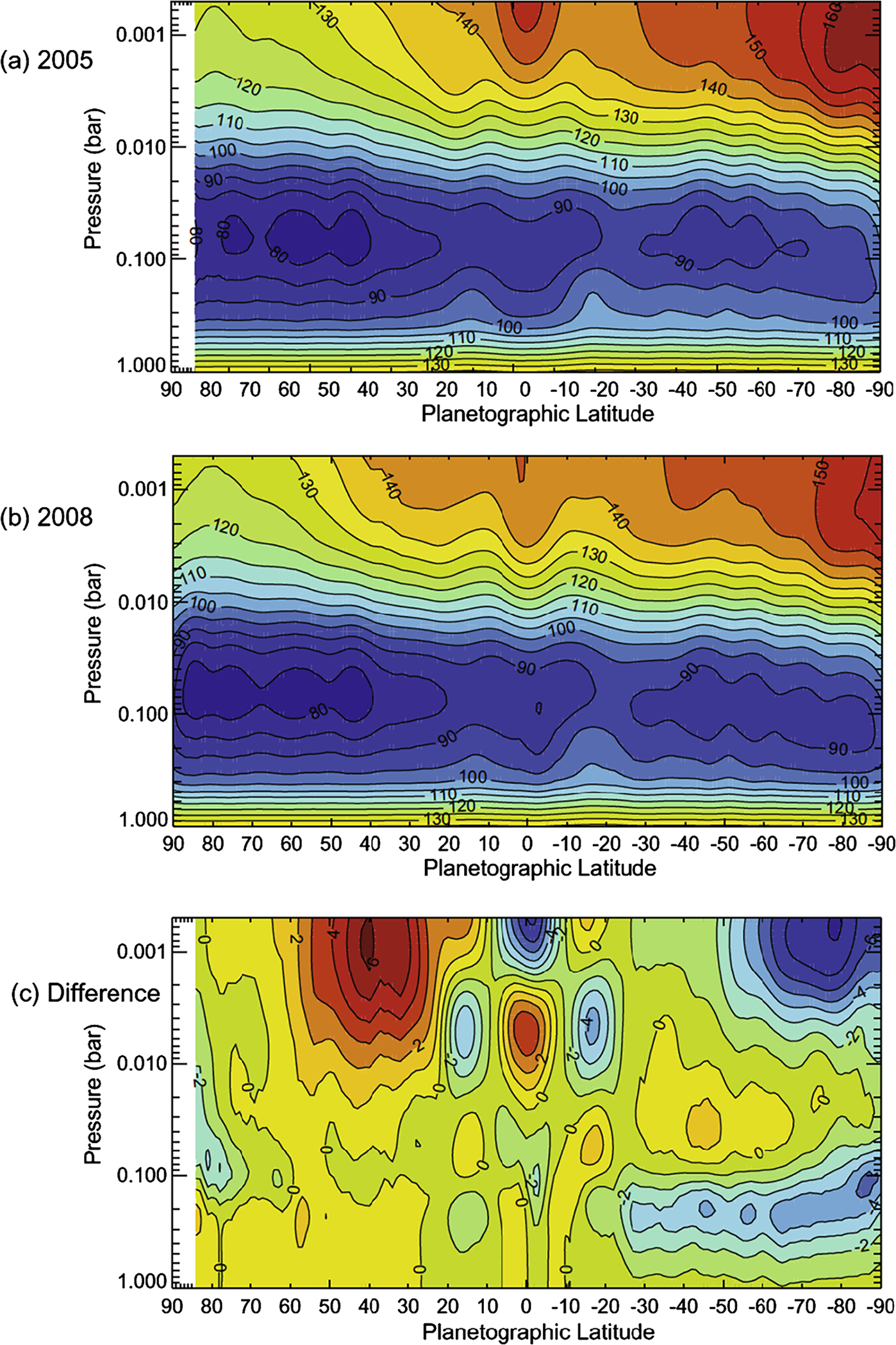
Temperature as a function of latitude and altitude from Cassini CIRS. In 2005 the season was mid-winter in the north, and in 2008 the season was late winter. Vernal equinox was August 10, 2009. The south is warmer than the north in both years, although most of the hemispheric contrast and most of the difference between 2005 and 2008 is confined to high altitudes (Fletcher et al. 2010)

Two opposing effects control the amplitude of seasonal variations on Saturn (Fig. 4). The obliquity is 26.73°, which is larger than that of Earth, and is tending to produce large seasonal variations in incident sunlight. These variations are augmented by the rings, which block sunlight from reaching large parts of the winter hemisphere. On the other hand, Saturn’s troposphere is massive, with clouds of water down to the 20-bar level, assuming the enrichment of water relative to solar composition is the same as that of methane, whose abundance can be measured spectroscopically. Further, the high specific heat of hydrogen gives the troposphere a huge thermal inertia, so the atmosphere tends to average over the seasonal cycle rather than respond to it. Seasonal averaging is reduced in the stratosphere (Fig. 4), because it has less thermal inertia than the troposphere. Seasonal change is reflected in the emitted power as a function of latitude (Li et al. 2015) as the northern hemisphere season changed from winter to late spring over the period 2004–2013 (Fig. 3).

Using Cassini CIRS data Fletcher et al. (2010) showed that the stratosphere has a greater range of temperatures than the troposphere, which is consistent with the greater thermal inertia of the latter. They point out that a photochemical haze develops in the spring/summer hemisphere (Fig. 1). Fletcher et al. (2015) observed polar cooling and depletion of acetylene in the southern hemisphere during fall and winter. Tropospheric temperature variations are small (Fletcher et al. 2016) and show a substantial seasonal lag time, as expected. These seasonal changes are superposed on a general upwelling in the tropics and downwelling at the poles, as revealed by the two states (ortho and para) of the H_2_ molecule (Fletcher et al. 2016, 2017). Sinclair et al. (2013) and Sylvestre et al. (2015) compare CIRS observations of C_2_H_2_, C_2_H_6_, C_3_H_8_ and temperature between 2005 and 2010 with a static photochemical model (Moses and Greathouse 2005) and find substantial effects of circulation and transport in addition to variations due to chemistry. Seasonal change of temperature, composition and aerosol is extensively reviewed by Fletcher et al. (2018c).

### 3.3 Zonal Jets and Temperatures

Winds are motions of the atmosphere relative to the rotating planet, and for Jupiter, Uranus and Neptune the wobble of the planet’s tilted magnetic field provides the planetary rotation rate. Saturn’s field is tilted less than 0.007° and therefore does not provide a physically-based reference frame (Cao et al. 2020). For locating cloud features, scientists use a period (10:39:24 s) that was defined following the Voyager encounters, knowing that the computed winds might be systematically too fast or too slow by over 100 ms^−1^.

**Fig. 5:**
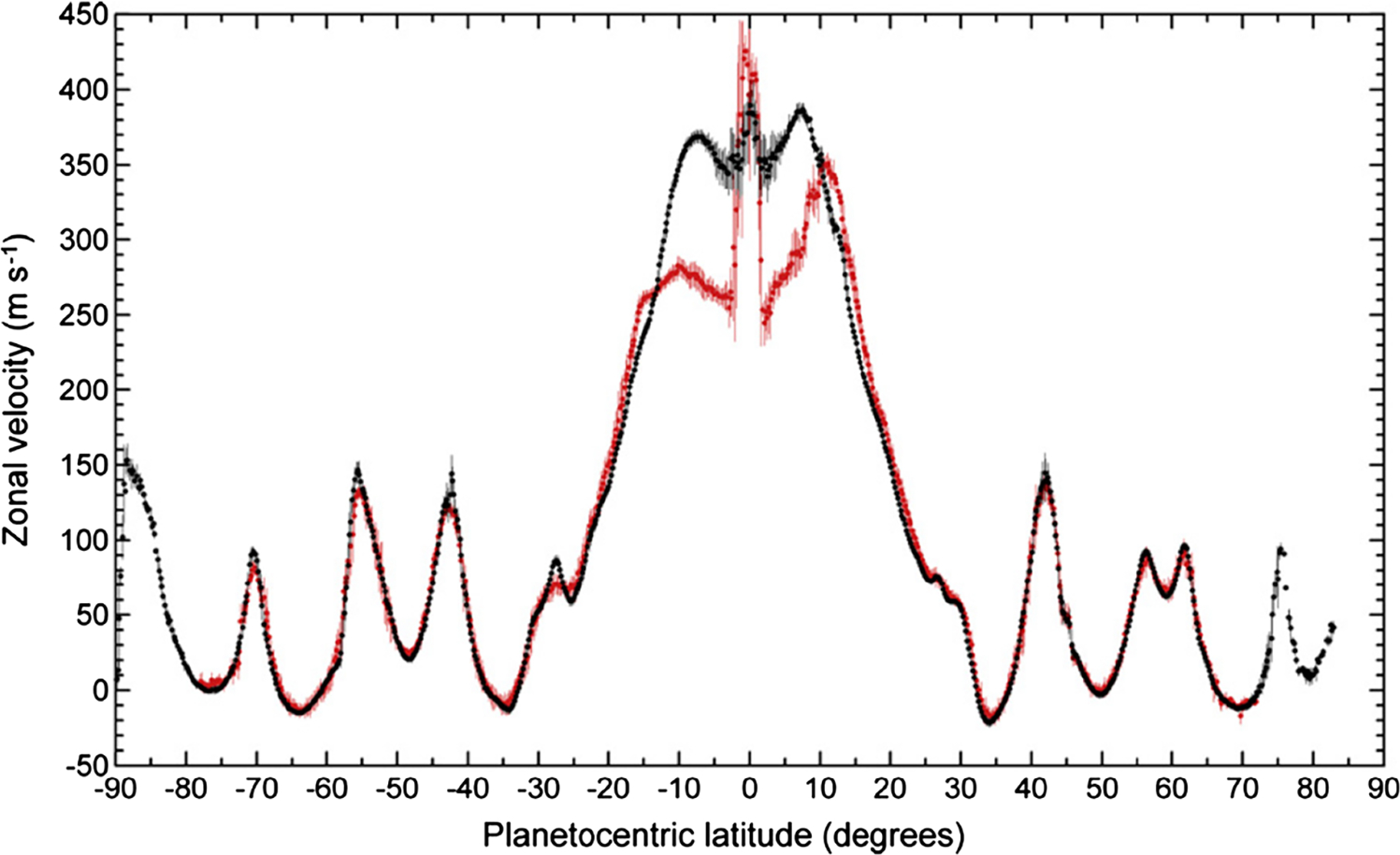
Atmospheric zonal velocity for Saturn from Cassini ISS. The black curve is data from the 350 to 700 mbar levels, and the red curve is data from 100 to 200 mbar. The differences are small except for a latitude band ±15° from the equator (Garcia-Melendo et al. 2011b)

Tracking small clouds in sequences of images is the principal way of measuring the winds (Fig. 5). ISS provided the first data at the start of the Cassini mission (Garcia-Melendo et al. 2011b; Porco et al. 2005; Vasavada et al. 2006) and a valuable comparison with Voyager 25 years earlier. VIMS can measure the winds at somewhat deeper levels than ISS. CIRS can measure the gradient of the wind with respect to altitude by measuring the gradient of temperature with respect to latitude and applying the thermal wind equation. Except at the equator, the mean zonal winds—the eastward winds averaged with respect to longitude—are remarkably steady. This conclusion follows, in part, by comparing the mean zonal winds during Voyager times with those measured by Cassini 25–30 years later (Antunano et al. 2015; Choi et al. 2009; Li et al. 2011, 2013; Vasavada et al. 2006). Images taken at wavelengths where methane is a strong absorber tend to sample higher altitudes than those where methane is transparent. Vertical shears show up in the eastward jets (Garcia-Melendo et al. 2009, 2011a, 2011b), which is consistent with the horizontal temperature gradient and the thermal wind equation

**Fig. 6:**
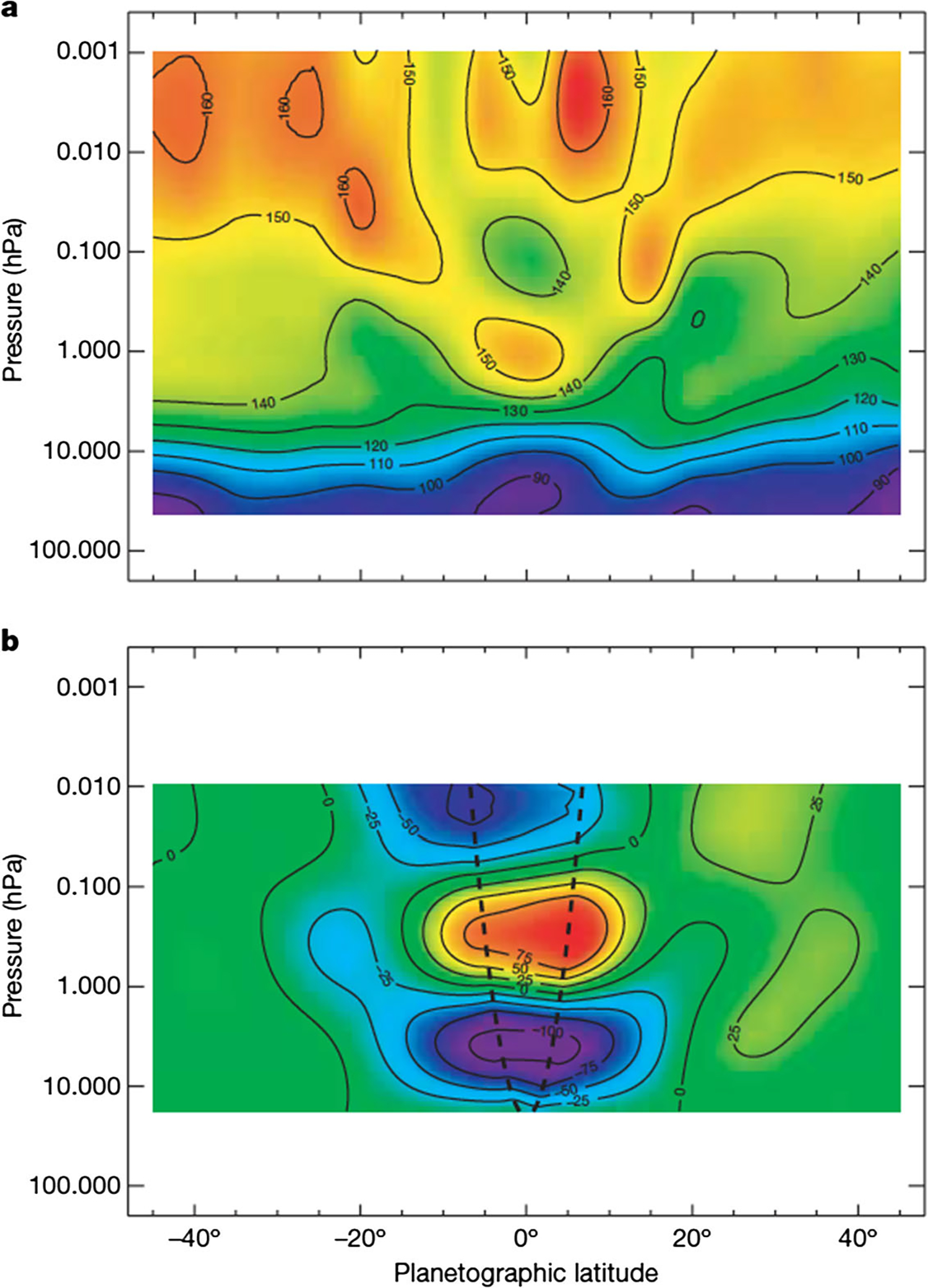
Oscillation of temperature (upper panel) and mean zonal wind (lower panel) at the equator from Cassini CIRS. The pattern moves down with time and has a ~15-year period (Orton et al. 2008; Fouchet et al. 2008; Guerlet et al. 2018). A similar pattern on Jupiter has a period of ~4 years (Orton et al. 1991). The Figure is from Fouchet et al. (2008)

At the equator, the speed of the zonal wind in the upper troposphere and stratosphere oscillates with a ~15-year period, which is half the Saturn year (Fouchet et al. 2008) (Fig. 6). In that sense, it is a semiannual oscillation. Since vertical shear in the zonal wind is related to horizontal gradients of temperature through the thermal wind equation, the oscillation reveals itself in the temperatures on either side of the equator. It shows up in the lower panel of Fig. 4 as the tripolar structure spanning the equator. Similar oscillations occur in the stratospheres of Earth and Jupiter. On Jupiter, the period is about four years. On Earth, there are two oscillations: the semi-annual oscillation (SAO), which has a period of six months, and the quasi-biennial oscillation (QBO), which has a period of about 22 months. The QBO is thought to be forced by waves propagating up from the troposphere, with feedback between the wind profile and the altitude where the waves deposit their zonal momentum. Downward propagation of the wind profile and the upward-propagating waves needed to support it have been extracted from CIRS limb observations (Guerlet et al. 2011, 2018).

The SAO period is the time between successive solstices, regardless of sign, and it could be forced by radiation. Sinclair et al. (2014) compare temperatures and gaseous abundances from Cassini with those from Voyager exactly one Saturn year later. They find changes, but most of them could be accounted for by a change in phase of the stratospheric oscillation, suggesting that the oscillation is only “quasi-semiannual”. For a review, see Read (2018).

In preparation for the Cassini encounter, Voyager and Hubble Space Telescope observations were used to document the variability of the zonal wind in the equatorial stratosphere (Perez-Hoyos and Sanchez-Lavega 2006; Sanchez-Lavega et al. 2000). Sanchez-Lavega et al. (2007) and Garcia-Melendo et al. (2010) used Cassini ISS images in the methane bands to further document the vertical wind shear and its temporal variability. Li et al. (2008) used Cassini CIRS data, where the oscillation is revealed in stratospheric temperatures, and Li et al. (2011) showed that the temperatures and cloud-tracked winds are consistent with the QBO oscillation mechanism. Schinder et al. (2011) used radio occultations by the Cassini spacecraft to measure temperature and showed that the zonal wind profile propagates downward as on Earth, consistent with the wave forcing mechanism of the QBO. Downward propagation also shows up in the thermal infrared maps based on CIRS data (Fouchet et al. 2008; Guerlet et al. 2011). Sanchez-Lavega et al. (2016) used ISS data to track individual features in the upper haze and clouds and further demonstrated the intense vertical shears and temporal variability.

A fundamental question dating back to Voyager days (Gierasch et al. 1986), for both Jupiter and Saturn, is why the zonal jets and the flow around the large ovals decay with height above the tops of the clouds. For Saturn this decay is seen, first, by tracking clouds in filters that sample different altitudes, like the winds within ±15° of the equator in Fig. 5 (Garcia-Melendo et al. 2009, 2011b). Second, the decay is inferred from the temperature gradients with respect to latitude—the spiky excursions in Fig. 3 and the little bumps and wiggles at mid latitudes in Fig. 4. These bumps and wiggles are not noise; the peak gradients are located at the latitudes of the zonal jets, and the thermal wind equation integrated upward from the cloud tops indicates that both the eastward jets and the westward jets are losing speed with altitude (Fletcher et al. 2008; Read et al. 2009b). The opposite behavior seems to occur from 2 bars to 300–500 mbar, as seen in Cassini VIMS images (Studwell et al. 2018). The temperature gradients could be forced by differential heating, or the decay of the jets could be forced by wave transport of momentum or both, since the two are linked by the thermal wind equation. Either way, the forcing has not been properly identified and the question remains open.

## 4 The Great Storm of 2010–2011

### 4.1 Storm Clouds and Convection

**Fig. 7:**
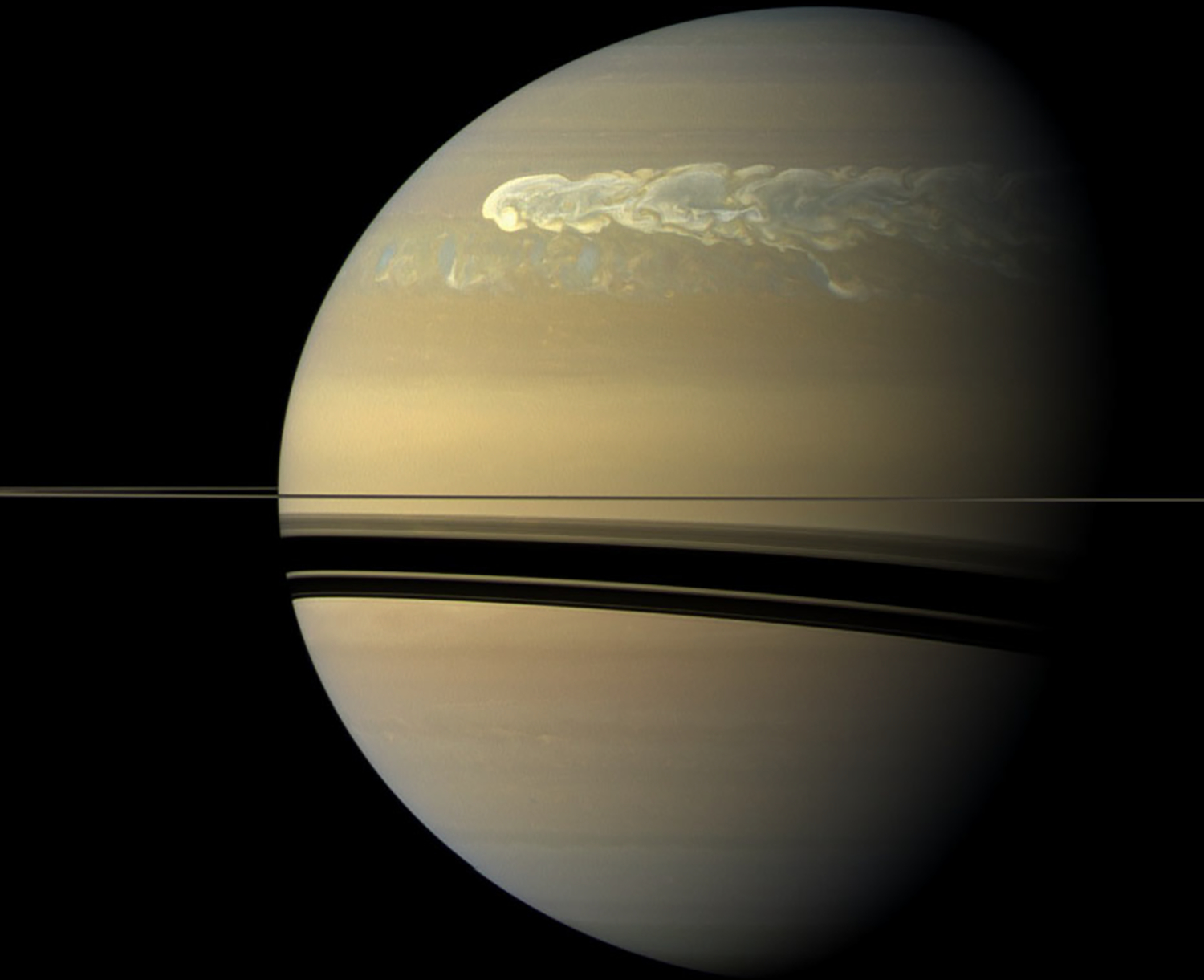
Saturn imaged by Cassini ISS on February 25, 2011. The great storm of 2010–2011 is clearly visible in a band centered at 30–40° latitude. Since its appearance on December 5, 2010, the head of the storm had drifted west and overtaken the tail (PIA 12826)

Saturn is prone to large-scale eruptions (Fig. 7). They start with a small cloud that grows rapidly during its first week and expands zonally, until after 2 or 3 months the disturbance has fully encircled the planet within its latitude band. The paper by Sanchez-Lavega (1994) is the definitive pre-Cassini review of these phenomena. He calls them Great White Spots, and he lists five of them before the Cassini era: 1876, 1903, 1933, 1960, and 1990. This gives an average periodicity of 28.5 Earth years, which is close to Saturn’s year of 29.46 Earth years. The corresponding latitude bands of these storms are 8±3°, 36±2°, 2±3°, 58±1°, and 12 ± 1°, all in the northern hemisphere. Whether the disturbances are in a unique class or just the largest in a continuous distribution of sizes is uncertain. Intermediate-scale storms do exist (Sanchez-Lavega et al. 2020a). And whether the disturbances are locked in phase to Saturn’s year is also uncertain, because within each latitude band the period is much longer than a Saturn year. And the great storm of 2010–2011 came 10 years too early to satisfy the annual cycle hypothesis.

**Fig. 8:**
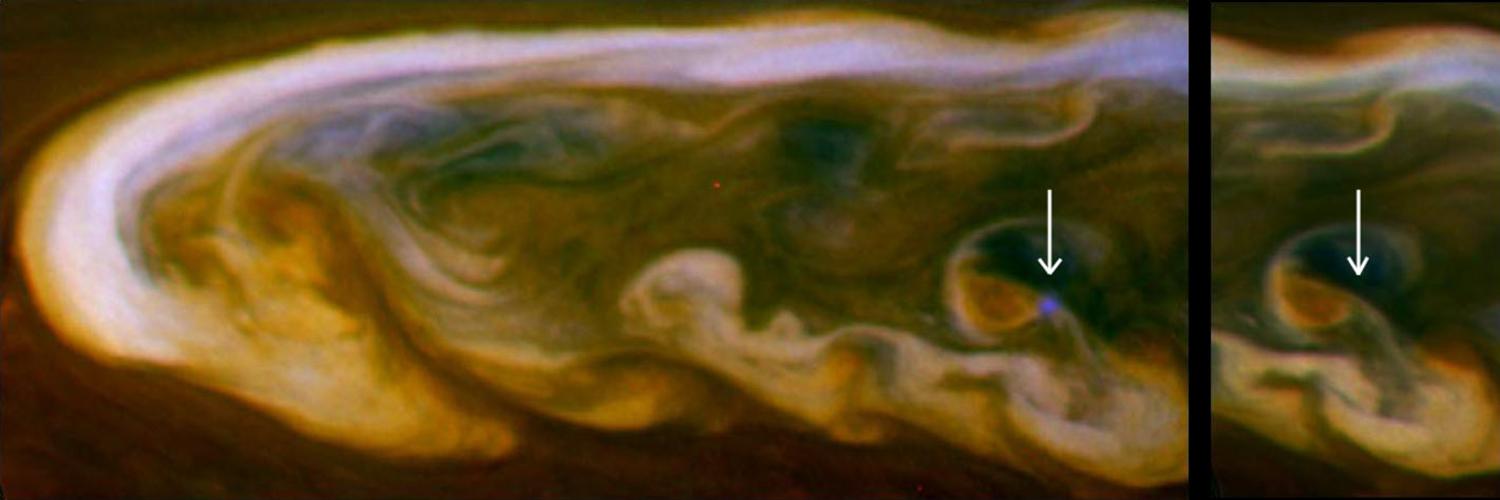
Lightning flash in Saturn’s great storm of 2010–2011. The color composite consists of three images taken in rapid succession at three different wavelengths. A lightning flash occurred while the blue-filtered image was taken, making a blue spot in the composite image, left. The same region was imaged 30 minutes later and did not see a lightning flash, right (PIA14921)

Saturn’s great storm of 2010–2011 was a singular event in Cassini’s 13-year tour of the Saturn system (Sanchez-Lavega et al. 2011, 2018). It began as a small (~1000 km) spot captured in a routine ISS image on December 5, 2010. Cassini Radio and Plasma Wave Spectrometer (RPWS) had been detecting lighting discharges (Fig. 8) from the storm for a few hours when the image was taken (Fischer et al. 2011). The lightning continued for the ~7 month lifetime of the storm as the westward-moving head left behind a tail that eventually wrapped around the planet. In its mature phase, the storm filled a latitude band 10,000 km wide that was centered at a planetocentric latitude of 35°. Amateur astronomers with Earth-based telescopes were following the storm within days of its appearance, and Cassini began systematic imaging in January 2011.

**Fig. 9:**
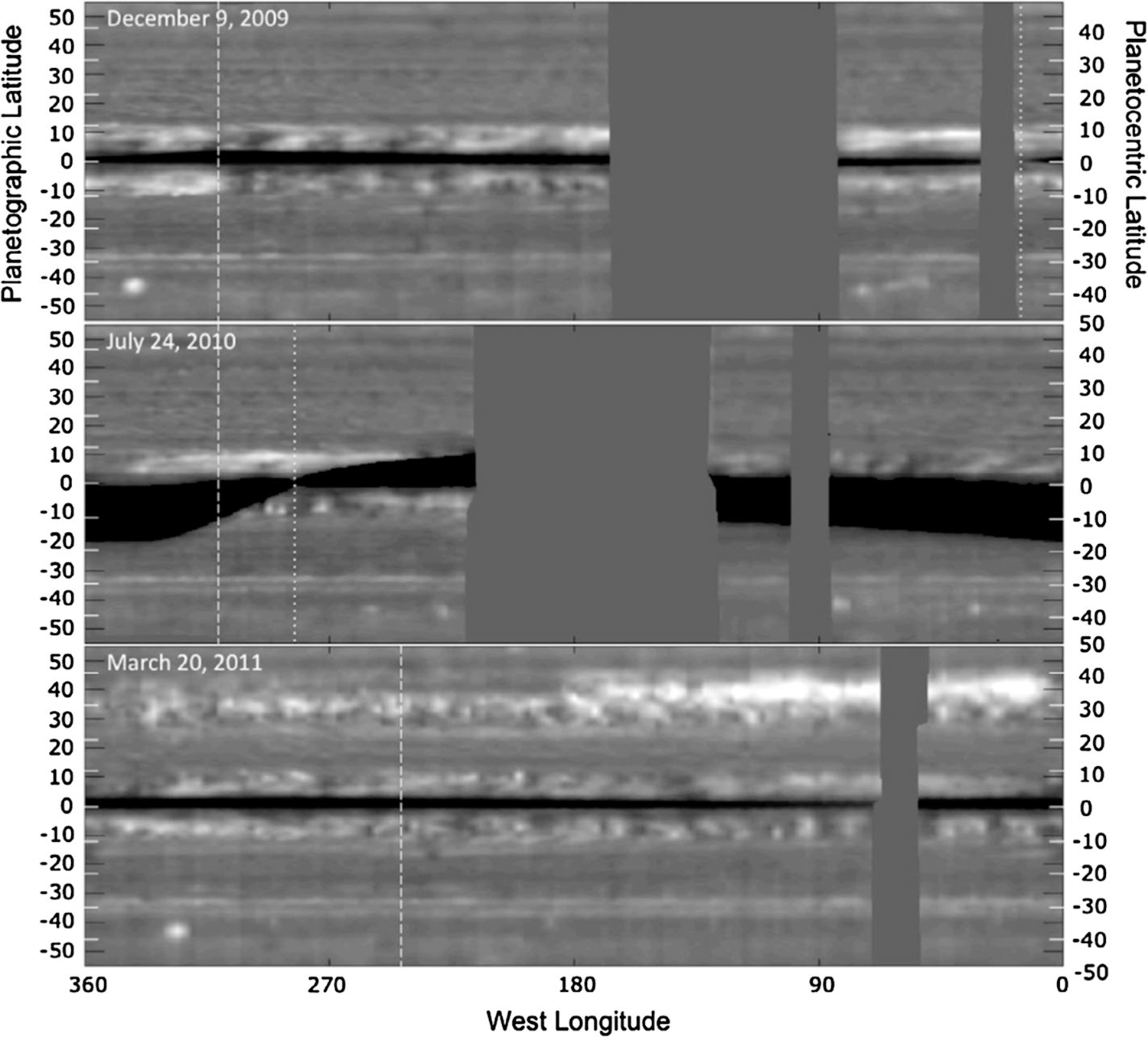
Cylindrical projection of Saturn’s thermal emission at 2.2 cm wavelength, obtained by the Cassini RADAR in passive (non-transmitting) mode. The dark band at the equator is due to the rings, which are colder than the planet itself. The top two panels show the planet before the great storm. The lower panel shows warm emission at the location of the storm. Ammonia is the principal absorber at 2.2 cm wavelength, so the warm emission is due to ammonia depletion allowing radiation from deeper levels to reach the detector (Janssen et al. 2013)

Visible-light images of clouds in the troposphere (Sanchez-Lavega et al. 2012; Sayanagi et al. 2013) showed that the head spawned large (10,000 km diameter) anticyclones that drifted off to the east and became part of the tail. Amateur astronomers provided nearly continuous coverage. The storm seems to have originated from a feature called the string of pearls, a train of cyclones at the latitude of the great storm (Sayanagi et al. 2014). Garcia-Melendo et al. (2013) are able to reproduce many of the observed morphological features of the storm with a prescribed heat source moving at a prescribed velocity. Li and Ingersoll (2015) present a theory that reproduces the observed drying (low mixing rate of ammonia) of the storm’s latitude band (Fig. 9) (Janssen et al. 2013; Laraia et al. 2013) as well as the multi-decadal interval between great storms. The latter is tied to mass loading, i.e., the stabilizing effect of water and ammonia condensation in a low molecular weight (H_2_ and He) atmosphere. Above a critical abundance of the condensates, the atmosphere becomes stable after a convective event, and the time needed to destabilize the atmosphere is decades. Based on the C/H ratios for Jupiter and Saturn, assuming the same enrichment factors relative to solar for O/H, Saturn is above the critical value and Jupiter is below it, which could explain why only Saturn has these great storms.

### 4.2 Chemical Tracers and Vertical Motion

**Fig. 10:**
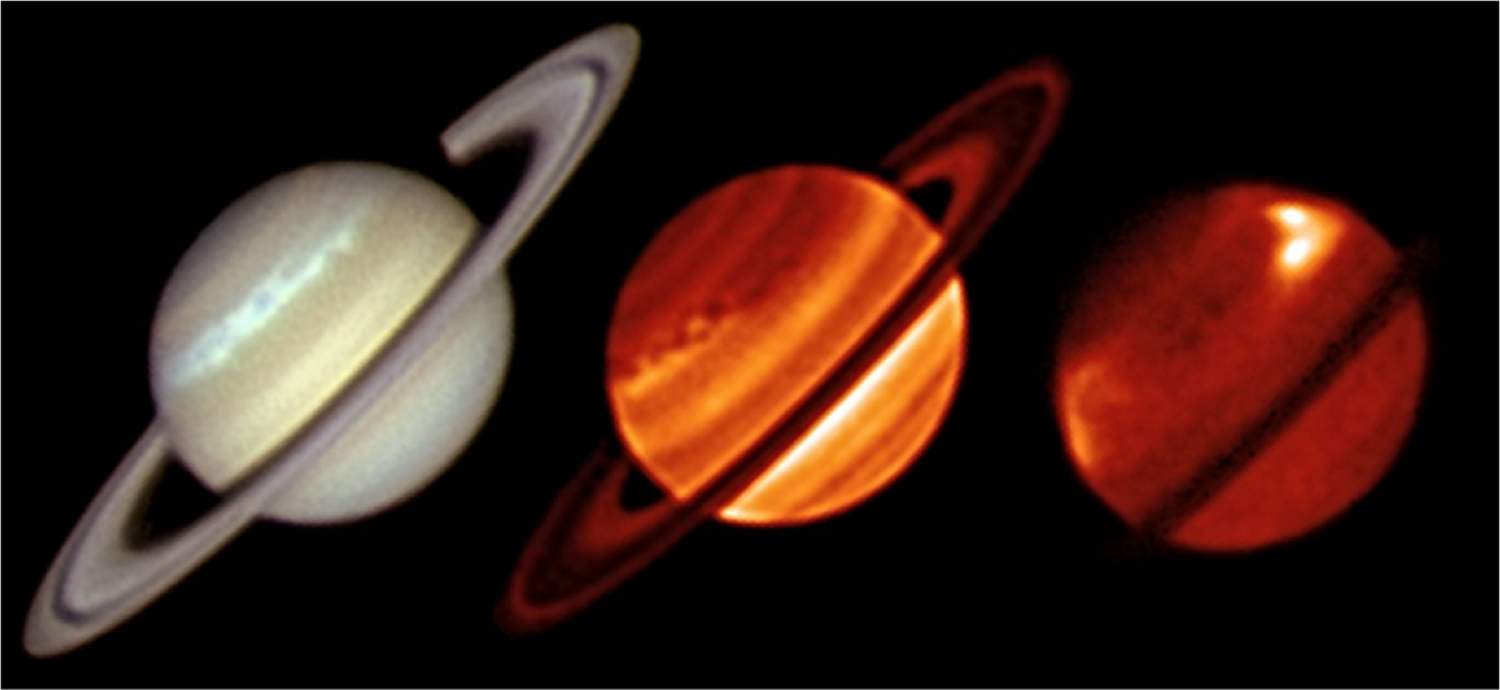
Visible light image, left, and infrared images sensitive to temperatures in the 200–500 mbar level, center, and the 1–10 mbar level, right, on January 19, 2011. The stratospheric beacon stands out in the right image. The visible light image was from the International Outer Planet Watch database (Hueso et al. 2010), and the infrared images were from the Very Large Telescope (VLT) in Chile (Fletcher et al. 2011b)

**Fig. 11:**
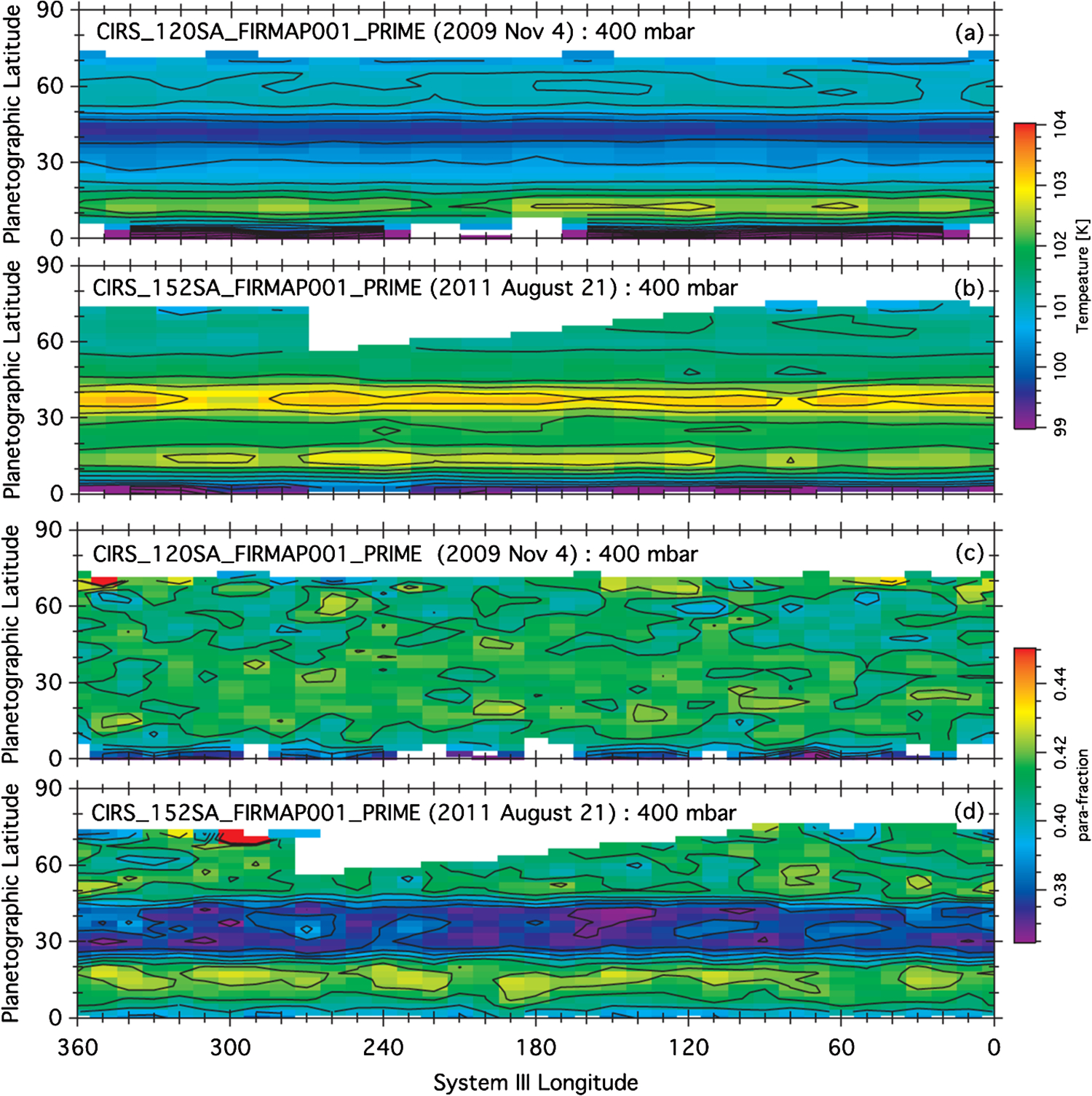
Cassini CIRS cylindrical projections showing evidence of air being dredged up from below. The top two panels show tropospheric temperature before and after the storm and the band from 30–40° planetographic has warmed by several degrees. The bottom two panels show the two chemical states (nuclear spins parallel and nuclear spins anti-parallel) of molecular hydrogen H_2_ before and after the great storm. The 30–40° band shows a low para fraction, which indicates that the air has risen from below cloud base (Achterberg et al. 2014)

Thermal infrared spectroscopy by Cassini CIRS revealed effects of the storm that penetrated into the stratosphere to the 1 mbar level (Fletcher et al. 2011b). Subsidence of air in the stably-stratified stratosphere produced beacons as much as 16 K warmer than their surroundings (Fig. 10). By May 5, 2011, the beacons had reached temperatures of 226 K, about 80 K warmer than the surrounding stratosphere (Fletcher et al. 2012; Fouchet et al. 2016). The storm produced a 100-fold increase of ethylene in the stratosphere near the beacon (Hesman et al. 2012). Photochemical models cannot explain this increase, so some dynamical mechanism must be at work (Cavalie et al. 2015; Moses et al. 2015). Temperatures in the troposphere at the latitude of the storm increased by 3 K, and the para fraction of H_2_ decreased by about 0.4, indicating warm air upwelling from below (Fig. 11) (Achterberg et al. 2014). The speed of the upwelling is uncertain partly because the time constant for approach of para H_2_ to equilibrium is uncertain and partly because the existence of a hydrogen dimer complicates the interpretation of the spectrum (Fletcher et al. 2018a).

The 2010–2011 storm seems to have affected clouds and haze, both at the latitude of the storm and farther away. Sromovsky et al. (2013) used VIMS spectra to study the composition of the cloud particles lofted by the storm, and found evidence of ammonia ice, water ice, and ammonium hydrosulfide. This is the first spectroscopic evidence of water ice in Saturn’s atmosphere and indicates upwelling from 200 km below the tops of the ammonia clouds. Further study of the aftermath of the storm reveals clearing of the ammonia cloud with some residual particles of other species (Sromovsky et al. 2016). According to an analysis of VIMS data, a residual haze layer persisted in the upper troposphere and lower stratosphere as well (Oliva et al. 2016). The great storm seems to have caused a 10 K warming of the middle atmosphere (0.5–5 mbar) at the equator, providing evidence of teleconnections between latitudes (Fletcher et al. 2017).

## 5 Vortices, Mean Flow and Eddies

### 5.1 North Polar Hexagon

**Fig. 12:**
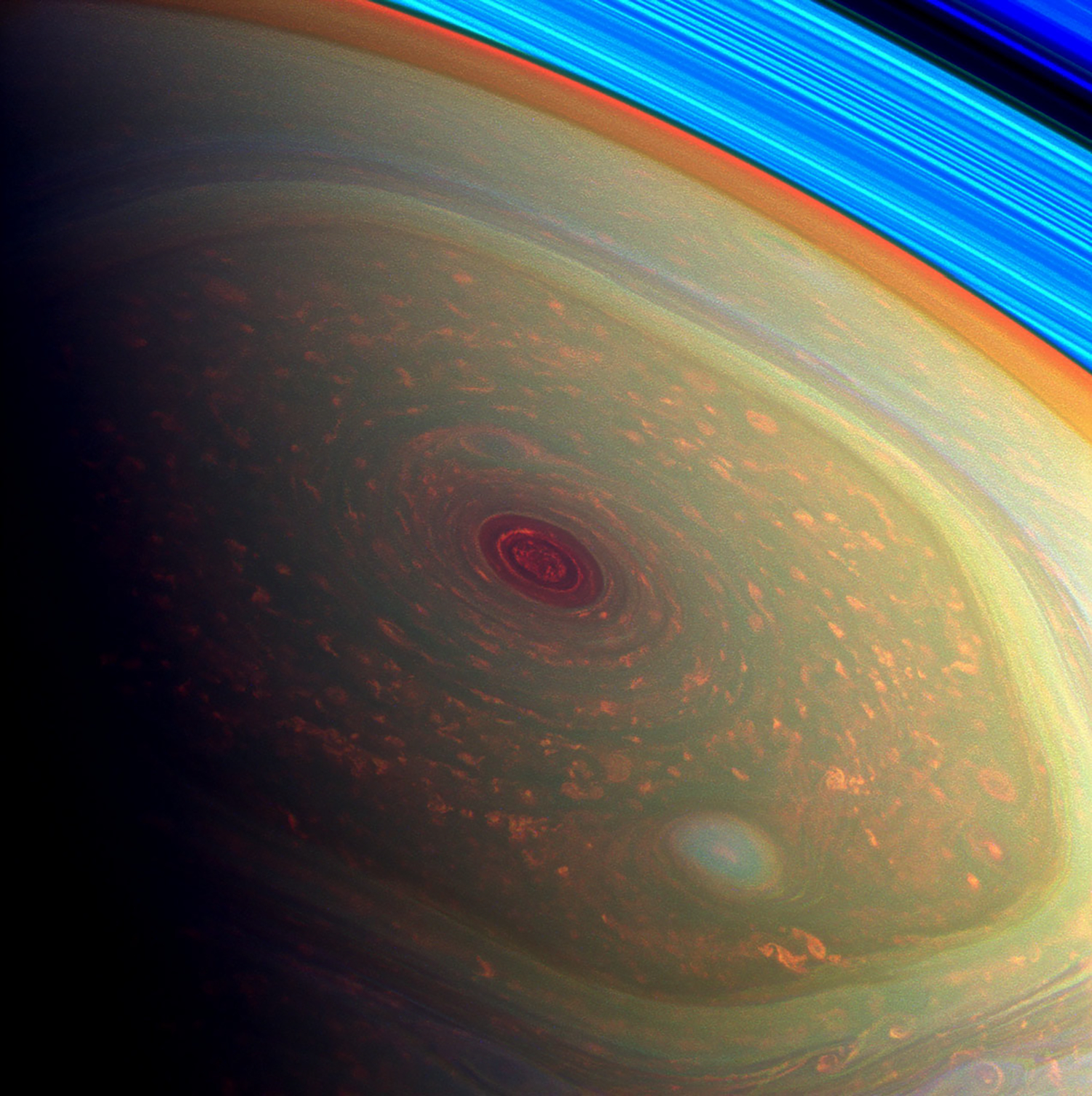
Saturn’s north polar hexagon and polar cyclone. This false color image was taken on November 27, 2012 by the Cassini ISS. The MT3 and MT2 images, which look dark due to absorption by methane gas in the atmosphere, are projected as blue and green, respectively. The CB2 image, which is not sensitive to methane absorption, is projected as red. The color balance is chosen to make the planet’s atmosphere look realistic. The rings look bright blue because there is no methane gas between the rings and the spacecraft. The red spot in the center extends from the pole to a latitude of 88–89°. It looks red in the false-color image because the clouds are deep and methane gas absorbs the sunlight before it can reflect off the clouds and reach the spacecraft. The spot is a cyclone with winds of ~100 ms^−1^ [PIA14946]

Saturn’s hexagon is a six-lobed meandering pattern in an eastward jet centered at 75° north latitude (Fig. 12). The excursions in latitude are approximately ±1°, which gives the structure its polygonal shape. Larger excursions would give it a more sinusoidal, wave-like shape. The hexagon was discovered in Voyager images taken in 1980 and was discovered again by Cassini VIMS, before the spring equinox when the North Pole was still in darkness (Baines et al. 2009b). Its effects extend into the stratosphere to altitudes of 0.5 mbar (Adriani et al. 2015; Fletcher et al. 2018b; Pryor et al. 2019; Sanz-Requena et al. 2018; Sanchez-Lavega et al. 2020a, 2020b). Based on ISS and VIMS imaging, the hexagon is a meandering jet whose 6-lobed pattern moves slowly or not at all relative to the nominal rotation rate for Saturn, which was defined following the Voyager encounter (Desch and Kaiser 1981). Small clouds within the jet move at ~125 ms^−1^ relative to the pattern. Thus the hexagon is like a road fixed in the Voyager reference frame, and the clouds in the jet are cars moving along the road. Based on the hexagon’s long-term stability, Sanchez-Lavega et al. (2014) argue that it is a deep-seated feature that could reveal the true rotation of the planet.

Each pole of Saturn is occupied by a single isolated cyclonic vortex with peak winds of 150 ms^−1^ (Dyudina et al. 2008, 2009; Sanchez-Lavega et al. 2006; Antunano et al. 2015; Liu et al. 2019). The latitude of the peak winds is 88.5° (Antunano et al. 2015), which means that the average relative vorticity within 1.5° of the poles is equal to 0.57 times the local planetary vorticity. Based on Cassini ISS, VIMS, and CIRS data, Dyudina et al. (2008) and Dyudina et al. (2009) interpreted the vortex as a hurricane-like eye with eyewall clouds at 88.5° extending 20–70 km above the clouds at the pole. Sromovsky and Fry (2019) argued that an abrupt change of optical thickness could mimic the apparent eyewalls. The region has a 4–7 K warm core extending from the upper troposphere into the stratosphere (Achterberg et al. 2018). The warm core and stable stratification imply downwelling, which is consistent with the observed low phosphine abundance in the core (Fletcher et al. 2008). Downwelling is also consistent with the near absence of high- and medium-level clouds in the eye (Sromovsky et al. 2020), although isolated convective clouds are present (Baines et al. 2018). Outside the eyewall are numerous anticyclonic vortices suggesting a convective origin (Dyudina et al. 2009). Sayanagi et al. (2017) compared the north and south polar cyclones and attributed the differences to seasonal effects. Polar phenomena are extensively reviewed by Sayanagi et al. (2018).

### 5.2 Eddy Mean-Flow Interaction

Giant planet atmospheres are a superb laboratory for studying the dynamics of rotating fluids. Cloud tracking provides estimates of the winds, and the lack of continents and oceans provides a simpler setting than on Earth. The Cassini mission advanced this field, not only by providing 13 years of observations of Saturn but also by providing a 3-month flyby of Jupiter with an upgraded suite of instruments compared with those on the Voyager flybys. The Jupiter observations are discussed in Sect. 8 of this paper. Here we focus on observations and models of the jets and vortices in Saturn’s atmosphere. Saturn’s atmospheric dynamics is extensively reviewed by Showman et al. (2018).

**Fig. 13:**
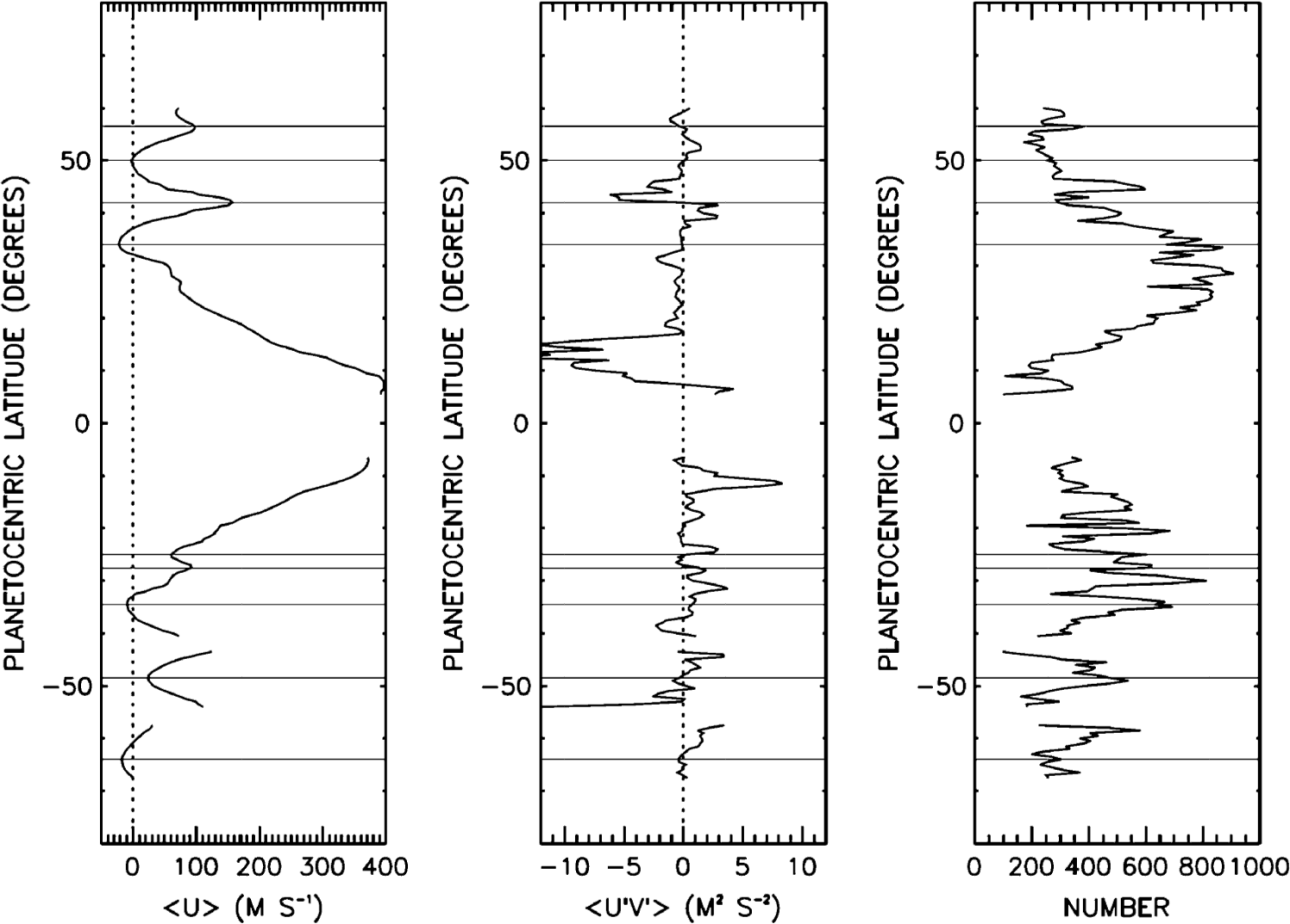
Eddy momentum transport for Saturn from Cassini ISS. The eastward and northward eddy winds u′ and v′ are the departures from the zonal means. Their product $\bar{u^{\prime }v^{\prime }}$ averaged over longitude and multiplied by density is the northward eddy flux of eastward momentum. The fact that this quantity has the same sign as $\partial \bar{u}/\partial y$, which is the increase of the mean eastward wind with latitude, says that the eddies are putting energy into the jets and not the reverse. The left panel is $\bar{u}$, the middle panel is $\bar{u^{\prime }v^{\prime }}$, and the right panel is the number of velocity measurements per 0.5° latitude bin (Del Genio and Barbara 2012)

The eddy momentum flux is a fundamental quantity. Eddies are the residuals after the time- and longitude-dependent mean winds $\bar{u}$ and $\bar{v}$ have been subtracted off. The eddy eastward wind is *u*′ and the eddy northward wind is *υ*′. By definition the means of *u*′ and *υ*′ are zero, but the mean of their product $\bar{u^{\prime }v^{\prime }}$ may be non-zero. Multiplied by the gas density, $\bar{u^{\prime }v^{\prime }}$ is the eddy momentum flux—the northward flux of eastward momentum. Using ISS data, Del Genio et al. (2007) and Del Genio and Barbara (2012) measured $\bar{u^{\prime }v^{\prime }}$ as a function of latitude (Fig. 13). The middle panel of Fig. 13 shows negative values of $\bar{u^{\prime }v^{\prime }}$ at 8–18° north latitude, indicating southward transport of eastward momentum by the eddies. The positive values at 8–18° south latitude indicate northward transport of eastward momentum. In other words, the eddies are subtracting eastward momentum from the westward winds beyond ±8–18° and adding it to the eastward winds within 8° of the equator. This seems counterintuitive, but it also occurs on Earth and Jupiter. The basic requirement is that the eddies have a source of energy that is distinct from the kinetic energy of the zonal jets.

If the eddy momentum flux were the only process acting, the jets could not be in equilibrium. To balance the eddies, there has to be an equatorward meridional flow ($\bar{v}<0$ in the north, $\bar{v}>0$ in the south), which carries low-angular momentum air to the equator. Thus air is approaching the equator from both sides. The net result is downwelling at the equator and upwelling where the meridional flows originate, i.e., in the ±8–18° latitude range. This picture applies in the troposphere and is consistent with the inferences drawn from the distributions of PH_3_ and AsH_3_ in Fig. 2.

Higher up, in the upper troposphere and stratosphere, other processes dominate. In the last paragraph of Sect. 3 we discussed the decay of zonal winds with height. Such decay requires a braking process—a force to the west acting on the eastward winds within ±8° of the equator (Fig. 5), and a force to the east acting on the westward jets farther from the equator. This force has not been identified, but it acts from the cloud tops up into the stratosphere. To balance it, there must be meridional flow, but that meridional flow must be diverging at the equator and converging between the eastward jet at the equator and the westward jets on either side of the equator. This leads to upwelling at the equator and downwelling on either side of the equator. This picture is qualitatively consistent with the inferences drawn from the NH_3_ distribution shown in Fig. 2 but is opposite to those drawn from PH_3_ and AsH_3_. Although the braking force has not been identified, it is based on the decay of zonal jets with height and is consistent with the stacked, oppositely-directed Hadley cells suggested by the tracer distributions. Zuchowski et al. (2009) show that a simple analytic model forced by Newtonian relaxation of heat and momentum, properly aligned, could give rise to the two-cell system.

Zonal jet stability is another important observation. Stability depends on the potential vorticity gradient (Antunano et al. 2019), which depends on vertical structure, both of temperature and wind, but a simple but relevant criterion is that the flow is stable if the curvature of the zonal wind profile with respect to latitude does not exceed *β*, which is twice the planet’s angular velocity times the cosine of latitude divided by the planetary radius. Interestingly, this stability criterion is violated on Saturn near the peaks of the westward jets, especially at high latitudes, indicating that the zonal jets could be unstable (Antunano et al. 2015; Read et al. 2009a). That does not mean the flow is unstable, however, as there are other stability criteria that involve vertical structure (Dowling 1995). Liao et al. (2007) argue that a statistical equilibrium occurs as the growing wave saturates. In their global vortex analysis, Trammell et al. (2014) find a correlation between the number of vortices and the westward jet peaks, implying at least some degree of instability at those latitudes.

Waves and vortices are another important feature of giant planet atmospheres. Saturn currently has nothing like the Great Red Spot, which has endured for over 100 years, but it has similar structures. Generally, anticyclones last longer than cyclones, but del Rio-Gaztelurrutia et al. (2010, 2018) document a cyclone that lasted for four years. For a comprehensive review of spots and vortices during the Cassini mission, see Hueso et al. (2020). Convective storms can generate waves that transport westward momentum away from their source regions, helping to accelerate eastward jets at the latitudes of the convection (Gunnarson et al. 2018; Liu and Schneider 2015; Sayanagi and Showman 2007). This acceleration must be balanced somewhere, and (Schneider and Liu 2009) argue that the balance occurs through magneto-hydrodynamic drag at 0.3 to 1.4 Mbar where the atmosphere becomes electrically conducting (Liu et al. 2014; Liu and Schneider 2015).

### 5.3 Modeling Studies

Saturn’s north polar hexagon at 75° latitude has inspired several modeling studies, both in the laboratory (Aguiar et al. 2010) and on the computer (Morales-Juberias et al. 2011, 2015; Rostami et al. 2017). Morales-Juberias et al. (2011, 2015) use the EPIC general circulation model with 20 vertical layers, and Rostami et al. (2017) use a shallow water model, which has only one layer. Both models start with an unstable zonal jet at the latitude of the hexagon, and with the right parameter settings the growing wave saturates as a hexagonal pattern that resembles the observed one. Morales-Juberias et al. (2015) get the best fit when the strength of the jet decreases with depth, and Rostami et al. (2017) get the best fit when they include a central cyclone within 2° of the pole. Central cyclones exist at both poles, and the main observable difference between north and south is that the southern jet is at a lower latitude than the northern one, 70.4°S vs. 75.8°N planetocentric (Antunano et al. 2015). The main unknown is the radius of deformation, which is related to the mean stratification within the weather layer—from the tops of the ammonia clouds to the base of the water cloud. Why there is only one hexagon is still uncertain.

Saturn’s ribbon at 47° latitude is a less dramatic relative of the hexagon (Sayanagi et al. 2010), since both seem to represent a steady meandering pattern on an eastward jet stream. The models do not explain why the conditions necessary for a long-lived meandering jet stream exist only at certain latitudes on Saturn or why there are no such jet streams on Jupiter. O’Neill et al. (2015, 2016) propose a model of Saturn’s polar cyclones in which convection at mid latitudes produces a vortex that is cyclonic at the bottom and anticyclonic at the top. The two halves separate, and the cyclonic vortex drifts to the pole and merges with other cyclonic vortices to make a single polar vortex. Brueshaber et al. (2019) report on one-layer shallow-water simulations of poleward drifting cyclonic vortices that merge to form polar vortices, and also reveal a mechanism that separateS the polar dynamical regimes of Jupiter, Saturn, and Uranus/Neptune. On the other hand, Antunano et al. (2018) report no meridional migration in the region north of the hexagon, and suggest that mergers do not contribute to the maintenance of the polar vortex. Sanz-Requena et al. (2018, 2019) describe the haze and cloud structure in the north pole and hexagon region.

Saturn’s great storm has also inspired modeling studies. One model uses an imposed heat pulse and studies its interaction with the ambient zonal flow (Garcia-Melendo and Sanchez-Lavega 2017). Another model studies how precipitation of water, because of its high molecular mass relative to hydrogen, can stabilize the atmosphere and inhibit convection for decades due to the long radiative time constant of Saturn’s atmosphere (Li and Ingersoll 2015). A third model uses a long-term numerical integration of moist convection in a giant planet atmosphere (Sugiyama et al. 2014). The simulations are conducted using a two-dimensional cloud-resolving model that explicitly represents the convective motions and microphysics of NH_3_, NH_4_SH, and H_2_O. It produces intermittent cumulonimbus activity. The time scale is ~60 days, although it is proportional to the water abundance and is therefore likely to be greater for Saturn than for Jupiter.

Numerical models of Saturn’s jets and vortices fall into two categories. One is the conventional general circulation model (GCM), which uses hydrostatic balance in the vertical and is valid for large horizontal scales and small vertical scales (Friedson and Moses 2012; Garcia-Melendo et al. 2007; Trammell et al. 2016; Spiga et al. 2020). The other is the fluid sphere model in which the vertical and horizontal scales are comparable and the flow takes place in thick spherical shells (Aurnou et al. 2008; Heimpel and Aurnou 2007; Liu et al. 2014). Computational limitations make it difficult to develop a single model that includes both small and large vertical scales, although the effort to do that is being made (Heimpel et al. 2016; Cuff and Heimpel 2018). One model (Yadav and Bloxham 2020) suggests that Saturn’s hexagon could extend down to where the magnetic field limits the flow speed by interacting with the electrically conducting fluid. More often, the practitioners divide into separate camps and work with different theoretical tools. The goal of these studies is to account for the observations—the number of belts and zones, their associated wind speeds, the stability and other properties of large vortices, and the direction of winds at the equator—prograde for Jupiter and Saturn and retrograde for Uranus and Neptune. The global distribution of temperature and outgoing longwave radiation with respect to latitude (Fig. 3), as well as the temperature gradients with respect to the zonal jets (Fig. 4) are another observable that the models could simulate. See the discussion at the end of Sect. 3.

### 5.4 Lightning

**Fig. 14:**
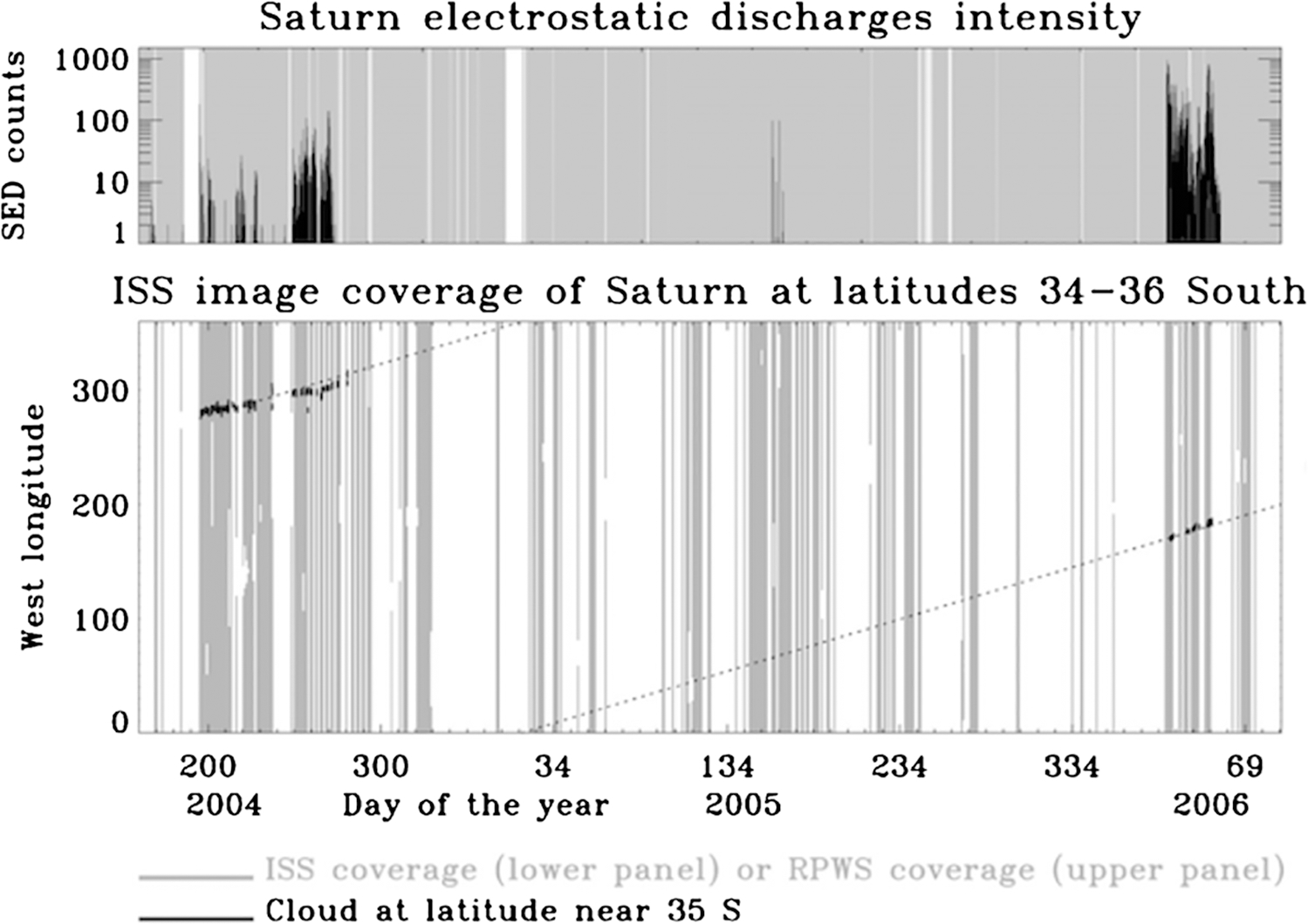
Saturn electrostatic discharge counts, SEDs, which are roughly equivalent to lightning strikes, over a 2-year period starting in 2004. The SEDs are detected by the RPWS instrument, which is ON continuously. The lower panel shows ISS coverage of latitudes −34° to −36°, where all the storms were occurring, and it is clear that storms were seen only when the RPWS was detecting SED’s (Dyudina et al. 2007)

The Cassini RPWS detects the radio waves from lightning at frequencies starting at 1.3 MHz and ranging up to 40 MHz (Fischer et al. 2006a; Fischer et al. 2006b; Fischer et al. 2007; Fischer et al. 2008). A single flash is called a Saturn Electrostatic Discharge (SED), and it lasts for less than the 35.2 ms integration time of the RPWS instrument (Fig. 14). Fundamentally, the duration of an SED is unknown (Farrell et al. 2007). An SED is not a whistler; it is a freely-propagating radio wave that follows a straight-line path from the source. Although the RPWS is listening virtually all the time, sometimes it detects nothing for months. SED activity is often from a single storm, with SEDs every few seconds for a 5-hour ON period followed by a 5-hour OFF period, when the storm is hidden behind Saturn. Often the storm itself is visible, both in ISS images (Dyudina et al. 2007) and in the images gathered by amateur astronomers at Earth. Criteria used to identify the storm are that it appeared within a day or two after the onset of SED activity and it was always on the side of the planet facing the spacecraft when the RPWS was detecting signals. Thus the number of lightning storms at any one time is likely to be either 0 or 1. In contrast, Earth has ~2000 lightning storms over the globe at all times, and Jupiter has dozens of storms at all times (Little et al. 1999).

Cassini ISS eventually detected the lightning flashes themselves (Dyudina et al. 2010), even on the day side despite the bright background of clouds in sunlight. The secret was to take many short exposures, since a short exposure reduces the background but not the lightning; it does reduce the probability of capturing a flash, so one needs to take many exposures. The diameter of the illuminated spot is about 200 km, indicating that the lightning is 125–250 km below cloud tops. This depth is above the liquid H_2_O–NH_3_ cloud and may be either in the NH_4_SH cloud or the H_2_O ice cloud.

The great northern storm of 2010–2011 was a copious lightning emitter (Dyudina et al. 2013; Fischer et al. 2011). The RPWS recorded flashes every 0.2 s. The optical energy per flash was about equal to that of the radio energy (Dyudina et al. 2013), although there is considerable uncertainty in both. The optical energy in single flashes ranged up to 8×10^9^ J. The flash rate and total power were hundreds of times greater than those of the smaller southern storms that had been appearing intermittently since the start of Cassini observations. Fischer et al. (2014) proposed that the northern storm could account for a change in the Saturn kilometric radiation (SKR) frequency through its effect on thermospheric winds.

The Saturn lightning results stimulated two reviews of planetary lightning (Yair et al. 2008; Yair 2012). Lightning is an agent of chemical change. Based on VIMS observations, Baines et al. (2009a) proposed that the dark and spectrally featureless clouds associated with giant planet thunderstorms represent small particles of elemental carbon. The particles are postulated to arise from atmospheric methane exposed to high temperatures during the lightning discharge, but Sromovsky et al. (2018) argue that the dark features are more likely regions of reduced optical depth. Dubrovin et al. (2014) study the effect of lightning on the lower ionosphere of Saturn. They find that H_3_^+^ ions are rapidly produced from the parent H_2_^+^ ion. On the other hand (Hurley et al. 2012a) searched for but found no correlation between acetylene and thunderstorm activity on Saturn. The Saturn results also stimulated lightning searches throughout the solar system and on exoplanets from Earth-based radio telescopes and from spacecraft (Hodosan et al. 2016; Zarka et al. 2008).

## 6 Interior Structure and Rotation

### 6.1 Magnetic and Atmospheric Periodicities

The periodic variation of the dynamo field has been used to estimate the interior rotation rates of Jupiter, Uranus, and Neptune. However, Saturn’s dynamo field axis is so closely aligned with its rotation axis that it has no measurable variation. Taking the misalignment of the other three giant planets, 10° for Jupiter, 60° for Uranus, and 47° for Neptune, the 0.007° upper bound for Saturn is highly improbable (1 chance in 10^6^ or 10^7^), given that the probability of the magnetic axis aligning with the rotation axis goes as the square of the angle between them (Dougherty 2017; Cao et al. 2020). The close alignment means that there is no detectable wobble in the field as the planet rotates and therefore no periodic signal with which to estimate the rotation period of the planet’s interior. The SKR radio emissions are tied to currents in the magnetosphere and ionosphere, and the period is variable from year to year (Fischer et al. 2014), which rules out a direct tie to the interior of Saturn.

There have been various attempts to use the atmospheric periods to estimate the interior rotation rate. They use the data in different ways, but they all derive a period that is near the midpoint of the atmospheric periods. Smith et al. (1982) use cloud tracking and choose a reference frame that minimizes the variance of the cloud-tracked zonal wind with respect to latitude, with 10:31:30 ±30 s as the result. Anderson and Schubert (2007) chose the reference frame that minimizes the measured shape of the 100 mbar surface from an equipotential, with 10:32:35 ±13 s as the result. Read et al. (2009b) use the reference frame in which the atmosphere is marginally stable with respect to Arnol’d’s second stability criterion, with 10:34:13 ±20 s as the result. Their method uses ISS and CIRS wind and temperature data to estimate potential vorticity in the range 2–270 mbar. The problem with these approaches is that they ignore the thermal wind equation, which allows vertical shear in the zonal winds. Thus the speed at depth might not match the average speed at the tops of the clouds where the winds and temperatures are measured. Read et al. (2009b) argue that the stability criterion depends on the speed of the longest Rossby waves, which are deeply rooted, perhaps extending to the base of the water cloud, but still the problem remains. For instance, the midpoint of the wind distribution at Earth’s upper troposphere would give a period between 22 hours and 23 hours for the rotation of the planet, because the average wind at the top of the troposphere is to the east.

The gravity field (Jacobson et al. 2006) also has information about the rotation rate of the interior (Hubbard et al. 2009). As with all planets, Saturn’s gravitational potential can be expanded into spherical harmonics, the leading term of which is the spherically symmetric potential −GM/R, followed by the zonal harmonics and the tesseral harmonics. The zonal harmonics are axially symmetric, and each term is proportional to a Legendre polynomial of degree *n* and a dimensionless amplitude factor J_*n*_. The tesseral harmonics are the longitudinally-varying part of the gravitational potential. Saturn is a fluid planet, and if it were in equilibrium only the zonal harmonics with even *n* would be present. The J_*n*_ reflect the response of the planet to its own rotation, so one could use them to infer the interior rotation rate if the interior structure were known. Conversely, one could infer the interior structure, e.g., core mass, metallic hydrogen, degree of helium separation, heavy elements in the molecular envelope, etc. if the rotation rate were known (Helled and Guillot 2013; Helled et al. 2015; Hubbard et al. 2009). Further information about the interior could come from the tidal Love number—the magnitude of the planet’s response to tidal forces (Lainey et al. 2017). The tidal observations are astrometric data on the orbits of Saturn’s moons spanning more than a century and include a large set of Cassini data. The study indicates significant tidal dissipation inside the planet. In all cases, an independent measure of the rotation rate is important for probing the internal structure.

### 6.2 Normal Modes and Gravity

**Fig. 15:**
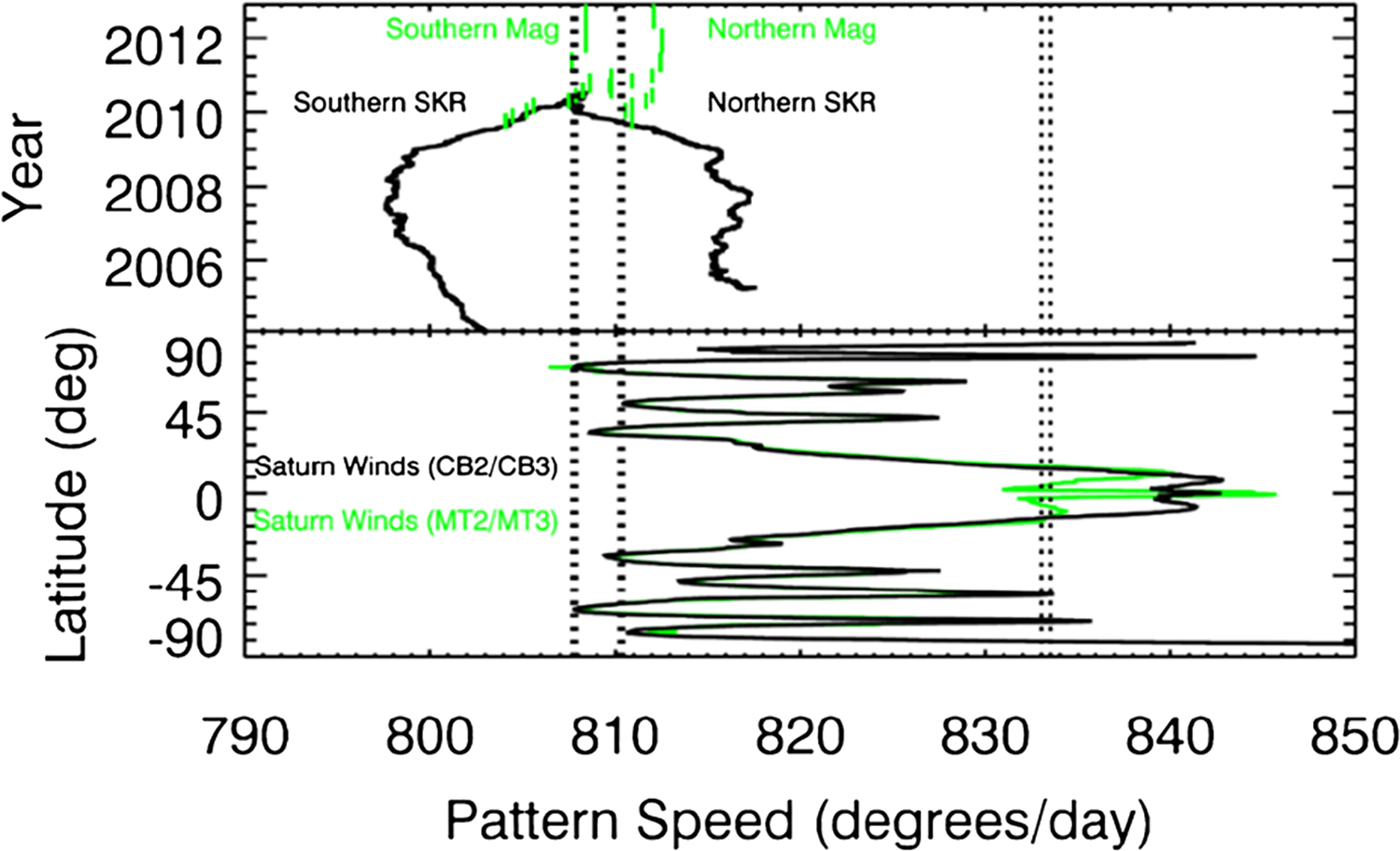
Ring seismology. The vertical dotted lines are the speeds of non-axisymmetric patterns in Saturn’s rings. Only the patterns that match the planet’s rotation are shown. They could be due to floating masses in the interior of Saturn. For comparison, periods of exterior magnetic fields, radio emissions, and clouds in the atmosphere are shown. Other patterns with speeds twice as fast are likely due to normal mode oscillations of the planet (Hedman and Nicholson 2014)

The tesseral harmonics have an effect on the rings, which are a sensitive seismometer for detecting the non-zonal gravity field (Hedman and Nicholson 2013, 2014). The harmonics are of two types. One type has pattern speeds ranging from 807 to 834 degrees per day, for which the corresponding periods are 10:42 m to 10:22 m, respectively (Fig. 15). These nearly bracket the periods derived from tracking the clouds at various latitudes in Saturn’s atmosphere, and could be due to non-zonal structures rotating with the planet. The large range of periods prevents improving on cloud tracking estimates alone.

The other types of tesseral harmonics have pattern speeds near five hours and are thought to be due to normal mode oscillations inside the planet. The precise frequencies of these normal modes depend both on the interior structure and the rotation rate. Parameters of the interior structure include the mass of the core, the helium mass fraction, the heavy-element mass fraction in the metallic and molecular envelopes, and the pressure at the metallic-molecular transition (Mankovich et al. 2019). For a given rotation rate, the interior parameters are adjusted to give the observed values of J_2_ and J_4_. The rotation rate is varied to give the best fit to the observed frequencies of the ~20 normal modes whose resonant effect on the ring orbits makes them detectable. The planet’s rotation affects the frequencies through the Coriolis and centrifugal forces and the ellipticity of level surfaces. The distribution of rotation periods resulting from a broad sample of interior models can be summarized as 10^h^ 33^m^ (Mankovich et al. 2019). The residuals of the fit do not exhibit any strong evidence of differential rotation inside the planet, but differential rotation cannot be ruled out. The rotation period is consistent with predictions by Militzer et al. (2019) who combined interior models, which were constrained by Cassini’s gravity measurements, with observations of the planet’s oblateness by the Voyager spacecraft. Based on this analysis, a rotation period of 10:33:34 ± 55 s is predicted for the planet’s deep interior.

The even zonal harmonics, especially *n* = 6 and above, can be used to detect differential rotation inside the planet (Galanti and Kaspi 2017; Kaspi 2013). This method is being used both with Cassini at Saturn and with Juno at Jupiter. The odd harmonics have been used only at Jupiter. The software to analyze these data was developed to be used on both planets (Galanti and Kaspi 2016; Galanti et al. 2017; Kaspi et al. 2016). Cassini produced its best gravity data during six dives between the planet and the innermost ring during the final five months of the mission (Iess et al. 2019). On these dives the spacecraft skimmed 2600–3900 km above the cloud tops while the radio link to Earth was monitored to determine the gravitational field of the planet and the mass of the rings. The gravity measurements yielded unexpectedly large values of the even zonal harmonics J_6_, J_8_ and J_10_. These values do not match any interior model that assumes uniform rotation and makes reasonable assumptions about the equation of state and helium rain (Iess et al. 2019). Galanti et al. 2019 confirmed that an acceptable solution can only be found for J_2_ and J_4_.

One can fit all the even harmonics J_2_-J_10_ with a model where unknown winds extend through the planet on cylinders. The best fit is where the atmosphere at the equator rotates 4% faster than the deep interior, but the models also require a region that rotates more slowly than the deep interior, which is surprising because it is not seen in the cloud tracking data. One can also fit the harmonics by assuming the observed cloud-top winds extend to a finite depth. The best fit is with a finite depth of 9000 km. Qualitatively the two results are the same, since the cloud-top winds are fastest at the equator. The depth of the winds for Saturn is 2–3 times deeper than that for Jupiter, but the difference is explained by the more rapid increase of electrical conductivity with depth on Jupiter. A comparison of the deep dynamics of Jupiter and Saturn in light of Juno and Cassini gravity measurements is presented by Kaspi et al. (2020). Whether magnetic drag suppresses differential rotation directly (Guillot et al. 2018) or indirectly through its effect on the stratification (Christensen et al. 2020) is an open question. Another open question is whether the measured odd zonal harmonics of the gravity field could be due to an extension of the cloud-level zonal winds, as seen on Jupiter by Juno (Kaspi et al. 2018). Kong et al. (2018) and Qin et al. (2020) claim that such an extension does not fit the data, and that the cloud-level winds are confined to a thin layer.

Once the gravity signal of the deep winds is adequately incorporated into models for Saturn’s interior, reasonable structures are obtained (Iess et al. 2019; Galanti et al. 2019; Movshovitz et al. 2020). The conditions of helium rain are compatible with predictions from ab initio computer simulations. Cores of heavy elements with 15–18 Earth masses are compatible with the core-accretion hypothesis, which requires an 8-Earth mass core to trigger run-away gas accretion. The enrichment of heavy elements in the planet’s molecular envelope of 1–3 times solar is lower than expected but still reasonable. This agreement with other sources of information contrasts with the persistent conundrum for Jupiter, for which the inferred heavy abundances are near or subsolar.

A puzzling feature of this analysis is that the same model of the gravitational field cannot fit all of the six passes in a combined, multi-arc orbital solution (Iess et al. 2019). The residuals represent unmodeled accelerations acting on Cassini over time scales of 20–60 min and could be due to time-varying tesseral harmonics. Convection in Saturn’s interior is one possible source. Normal modes varying in amplitude and frequency is another. The data were eventually fit by assuming random accelerations of 10 minute duration acting within ±1 hour from pericenter (Iess et al. 2019). The hope is that these high-frequency accelerations do not significantly affect the determination of the even harmonics J_2_-J_10_.

The amplitudes of the normal modes are a separate issue than the normal mode frequencies. Ordinary turbulent convection as on the Sun (Goldreich and Keeley 1977) falls short by at least six orders of magnitude (Markham and Stevenson 2018) because the kinetic energy is so low. Moist convection apparently has enough thermal energy to drive the modes, should it be available to drive them, but the available kinetic energy falls short by two orders of magnitude (Dederick et al. 2018). Rock storms are an intriguing candidate because their expected energy is greater than for conventional water storms (Markham and Stevenson 2018).

## 7 Upper Atmosphere Physics and Chemistry

### 7.1 Chemical Tracers and Ring Rain

The stratosphere entered in the discussion in the sections entitled composition and chemistry, seasonal variations, and zonal jets and temperatures. To reduce the overlap between those sections and this one, we focus here on theoretical models and on the parts of the atmosphere above the stratosphere. The entire subject, upper atmosphere and ionosphere, is reviewed in the book chapter by Nagy et al. (2009). Species like C_2_H_2_ and C_2_H_6_ have chemical lifetimes in the stratosphere that are comparable to a season on Saturn, so they can be used as tracers of the meridional circulation and its annual reversal from north to south. At the equator the zonal winds in the stratosphere exhibit a 15-year oscillation that is accompanied by an oscillation of the temperature distribution symmetric about the equator to maintain thermal wind balance.

The simplest chemical model is a diurnally averaged 1-D photochemical model with eddy diffusion chosen to represent dynamical processes. Moses et al. (2000a) developed a model that couples hydrocarbon and oxygen photochemistry, molecular and eddy diffusion, radiative transfer, and condensation to better constrain the chemical species and to identify the important physical processes that control the abundances. Moses et al. (2000b) consider the role of oxygen by comparing their model results with the observed abundances of H_2_O and CO_2_. They find that an external source of oxygen is necessary, and they estimate its magnitude. Ring particle diffusion and interplanetary dust are mentioned as likely sources (Moses and Poppe 2017).

During Cassini’s final orbits, the spacecraft flew between Saturn’s upper atmosphere and its innermost ring, the D ring. The Ion Neutral Mass Spectrometer (INMS) identified CH_4_, CO_2_, CO, N_2_, H_2_O, NH_3_, and organics in the ring material. The INMS estimate of >70% non-icy material exceeded remote sensing estimates and is still an open question (Miller et al. 2020). One source of nanograin particles is Saturn’s extended atmosphere extending into the innermost ring (Perry et al. 2018). Another source is electrostatic charging, which could lead to ejection of nanograins out of the ring plane and into the atmosphere by the electromagnetic force (Ip et al. 2016). Further evidence of ring rain is seen in enhanced emissions at latitudes tied to magnetic field lines that cross the equatorial plane at the locations of the inner edges of the A-ring and B-ring and at the orbit of Enceladus (O’Donoghue et al. 2013, 2017).

**Fig. 16:**
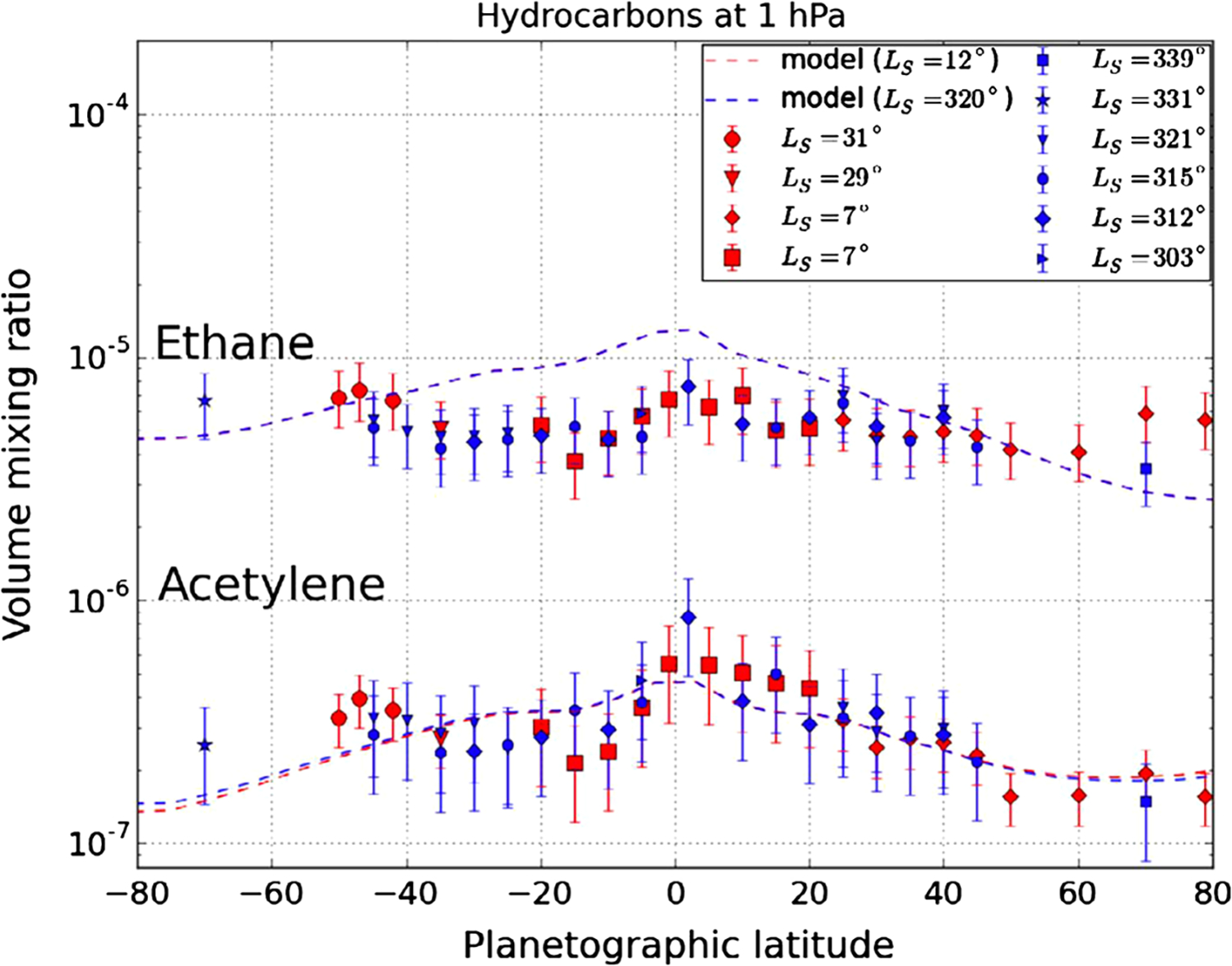
Hydrocarbons in the stratosphere at 1 mbar pressure. L_*s*_ = 0° and L_*s*_ = 360° are the first day of northern spring on successive years. The dashed lines are the results of a 1D photochemical model with seasons (Moses and Greathouse 2005). Ring shadow is included in the model, which has vertical mixing of species but no meridional circulation. The data are from the CIRS instrument on Cassini (Sylvestre et al. 2015)

Spatial and temporal variations in the mixing ratios of chemically reactive species hold clues to the circulation. Figure 16 shows the mixing ratios of C_2_H_6_ and C_2_H_2_ in the stratosphere just before and just after northern spring equinox (blue and red points, respectively). These hydrocarbons are produced as a result of methane photolysis at 0.1 Pa pressure levels, and they revert to much lower values by photochemical reactions at deeper levels, including 1 hPa where the mixing ratios are shown. Departures from photochemical equilibrium are thought to be due to a meridional circulation. The photochemical loss time of C_2_H_2_ is 3 years and that of C_2_H_2_ is 600 years, and the turnover time of the stratosphere at the 1 hPa level is between the two loss times (Guerlet et al. 2009; Sylvestre et al. 2015).

Hesman et al. (2009) compare the output of the static photochemical model with observations of C_2_H_2_ and C_2_H_6_, and they too find evidence of a meridional circulation. Guerlet et al. (2014) compare the temperatures computed from a radiative equilibrium model with temperatures observed by Cassini CIRS, and they find evidence that other processes, presumably related to dynamics, control Saturn’s stratospheric thermal structure. Teanby et al. (2006) place new upper limits on halides in Saturn’s stratosphere, but they advise that the abundances in the troposphere could be much larger.

**Fig. 17:**
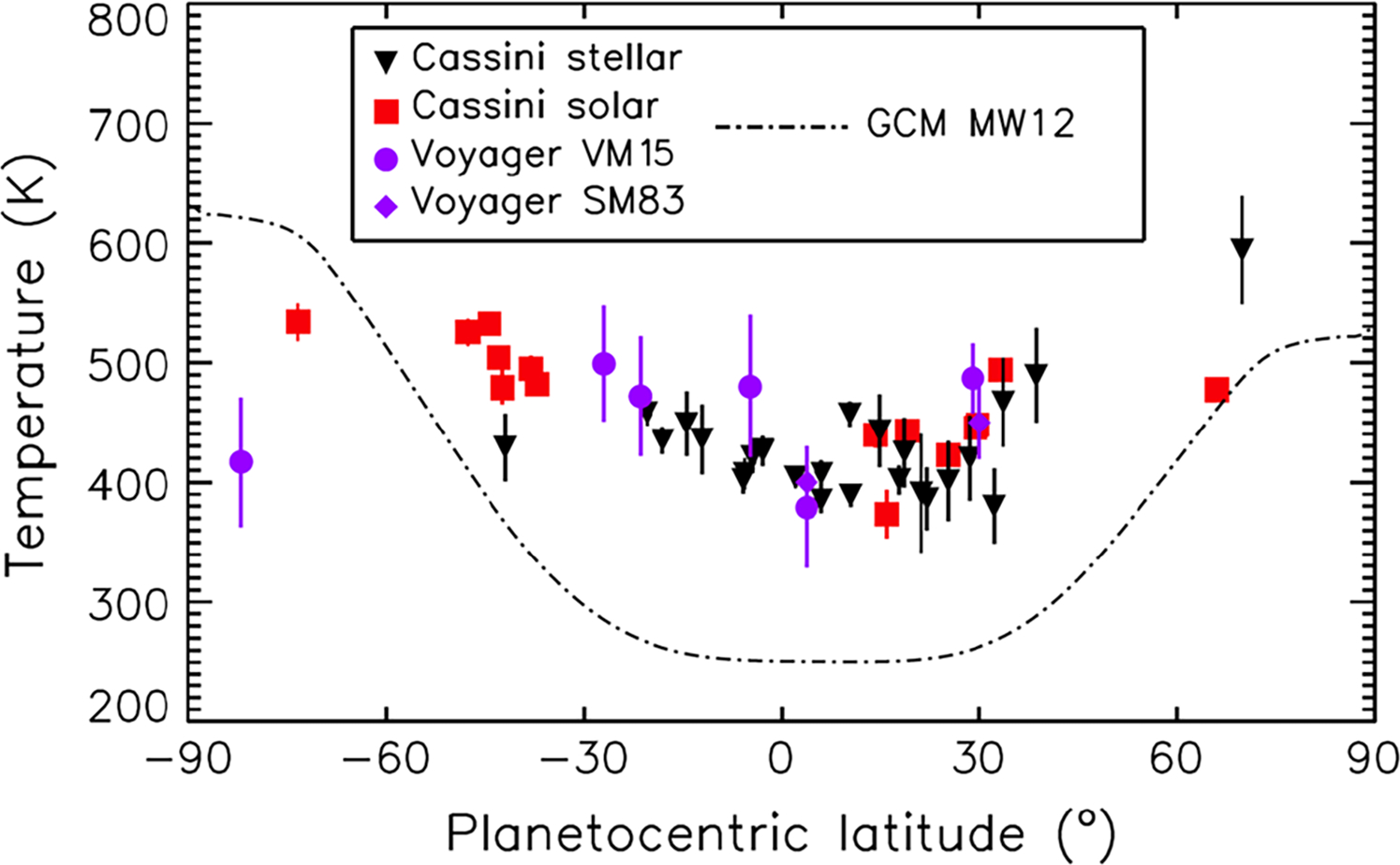
Comparison of Cassini UVIS observations with Voyager observations and with a 3-D general circulation model. The model result is represented by the smooth dot-dashed line, and it is significantly low (colder) equatorward of ±60° in both hemispheres. The problem is that the air from the Polar Regions, heated by auroral currents, cools before it reaches the lower latitudes. What keeps it warm is an ongoing mystery (Koskinen et al. 2015)

Above the stratosphere, diffusive separation takes over and each gas’s density falls off with altitude at its own individual scale height, which depends on its molecular weight. The altitude of the transition is called the homopause. Below the homopause, one finds gaseous hydrocarbons ranging in molecular weight at least up to benzene (Guerlet et al. 2015; Koskinen et al. 2016; Vervack and Moses 2015). These hydrocarbons are the source of stratospheric haze particles at non-auroral latitudes (Moses et al. 2000a; Kim et al. 2012). Above the homopause the major constituent is H_2_, which continues into the exosphere where the molecules don’t collide with each other except at the exobase (Koskinen et al. 2013). Atomic hydrogen H is also present (Koskinen et al. 2020). Its abundance is generally consistent with models based on photochemistry and transport. The relatively high (380 K to 590 K) exospheric temperature at low to mid latitudes is a long-standing mystery (Shemansky and Liu 2012; Stallard et al. 2012b). Despite the changing seasons, the high temperatures have persisted from the period of the Voyager flybys through the Cassini era (Koskinen et al. 2015). High temperatures do occur in the auroral zones, but dynamical models strongly suggest that the upper atmosphere is geostrophically balanced and cannot convey the high-temperature air to lower latitudes (Fig. 17). One possibility is that atmospheric gravity waves, which are often overlooked in models, could supply enough torque to overcome the Coriolis force and allow enough meridional overturning to warm the equatorial thermosphere (Mueller-Wodarg et al. 2019; Brown et al. 2020).

The first in situ measurements of the equatorial thermosphere occurred during the final month of the Cassini mission, when the spacecraft skimmed through the upper atmosphere on four periapses and also during the final plunge. The Ion Neutral Mass Spectrometer (INMS) measured the profiles of temperature and composition (Yelle et al. 2018). The best fit to the exospheric temperature is in the range 340 to 370 K, with a value during the final plunge of 354 K. The helium profiles are consistent with diffusive equilibrium, but the methane profiles are not. Even well above the homopause, the CH_4_/H_2_ ratio is nearly constant with height. This points to an external source, the most likely being Saturn’s rings (Yelle et al. 2018).

The aurora is caused by precipitating electrons striking the upper atmosphere. The source of the electrons is in the magnetosphere, and the subject is covered mainly in the section entitled Magnetospheres and Plasma Science (MAPS) Discipline Science Results. Some of the questions involve the energy and source of the electrons, how deep they penetrate into the atmosphere, and what effects they have on the temperatures, chemistry, and electromagnetic emissions associated with the aurora.

The aurora is observed in the ultraviolet (Gerard et al. 2013; Gustin et al. 2009, 2010, 2017), visible (Dyudina et al. 2016), infrared (Melin et al. 2011, 2016; O’Donoghue et al. 2014; Stallard et al. 2012a), and radio wavelengths (Lamy et al. 2009, 2013; Ye et al. 2016). The currents associated with the electrons can be observed when the spacecraft flies through an auroral flux tube (Bunce et al. 2014). And although the magnetosphere of Saturn is much less energetic than that of Jupiter, the footprint of the Enceladus flux tube has detectable emissions (Pryor et al. 2011), though not so bright as those of the Jovian moons: The geysers of Enceladus are weaker than the volcanoes of Io, and the magnetic field of Saturn is weaker than the magnetic field of Jupiter.

For a short, broad-brush summary of the magnetosphere, ionosphere, and atmosphere, we recommend Gombosi and Ingersoll (2010). This science objective is captured in detail in the Magnetospheres and Plasma Science (MAPS) Discipline Science Results under the following Cassini science objectives: Conduct in situ studies of Saturn’s ionosphere and inner radiation belt, investigate magnetospheric periodicities, their coupling to the ionosphere and how the SKR periods are imposed from close to the planet (3–5 R_S_) out to the deep tail, determine the coupling between Saturn’s rings and ionosphere, determine the temporal variability of Enceladus’ plume (relevant to the Enceladus auroral footprint).

## 8 Jupiter Science Results

### 8.1 Winds, Eddies and Long-Lived Ovals

**Fig. 18:**
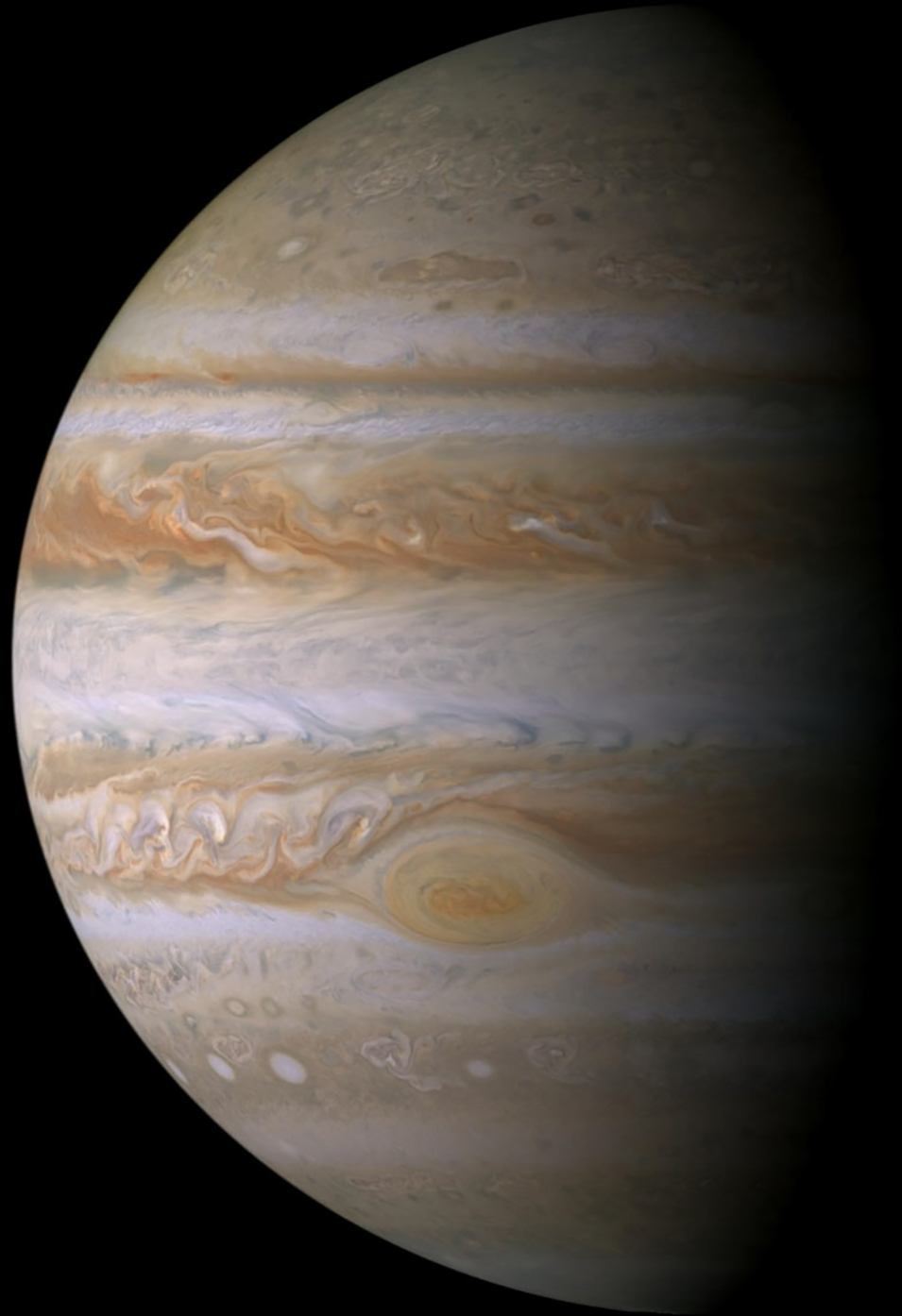
The highest-resolution, full-disk color mosaic of Jupiter ever taken. Jupiter more than filled the field of view of the ISS, so the mosaic was assembled from over 30 individual images, allowing for the planet’s rotation as the images were taken [PIA04866]

As it approached and flew past Jupiter in late 2000, Cassini provided three months of valuable data about the atmosphere. It is a photogenic planet; the colorful clouds provide ideal tracers of the winds (Fig. 18). The light and dark bands—the zones and belts—and their associated jet streams have been observed from Earth for more than 100 years. The Great Red Spot (GRS) and its smaller cousins have been observed for just as long. Thus Jupiter is an atmospheric dynamics laboratory, where dynamical phenomena like waves, jets, eddies, and vortices can be studied without the complications of continents, oceans, and large seasonal swings. A 70-day movie shows the clouds in motion at all longitudes and latitudes up to within ~10 degrees of the poles. That movie in cylindrical and polar projections is PIA03452, PIA03453, and PIA03454, and is available at https://photojournal.jpl.nasa.gov/.

The GRS is at least 150 years old, although it could be much older. The three white ovals to the south of the GRS formed in the late 1930s and merged into one oval named BA in the late 1990s. All of these features are anticyclones with circumferential winds greater than 100 ms^−1^. They drift slowly in longitude but stay fixed in latitude. Combined with Hubble Space Telescope and Galileo data, Cassini data were used to show that the GRS shrank by 15% from 1996 to 2006, both in terms of its visible appearance and its ring of circumferential winds (Asay-Davis et al. 2009; Shetty and Marcus 2010).

The white ovals occupied a single latitude band with anticyclonic vorticity. They avoided each other for 60 years because they were separated by cyclonic regions encroaching into the band from the equatorward side (Youssef and Marcus 2003). When the cyclonic regions got pushed out, the ovals merged. Peak velocities around oval BA remained steady while the color changed from white to red several years later. Using different techniques to measure winds and different definitions of the oval’s edge, different groups have documented small changes in the properties of BA over similar time periods (Hueso et al. 2009; Choi et al. 2010; Sussman et al. 2010). The combination of Cassini ISS data and Hubble methane band data was used to understand the changes in vertical cloud structure as BA changed from white to red (Wong et al. 2011). Using ISS data from the Cassini flyby (Li et al. 2004; Choi et al. 2013) and amateur telescopic data (Rogers et al. 2006), several authors have documented the life cycles—formation, shape, lifetime, mergers—of the more numerous smaller spots on Jupiter.

**Fig. 19:**
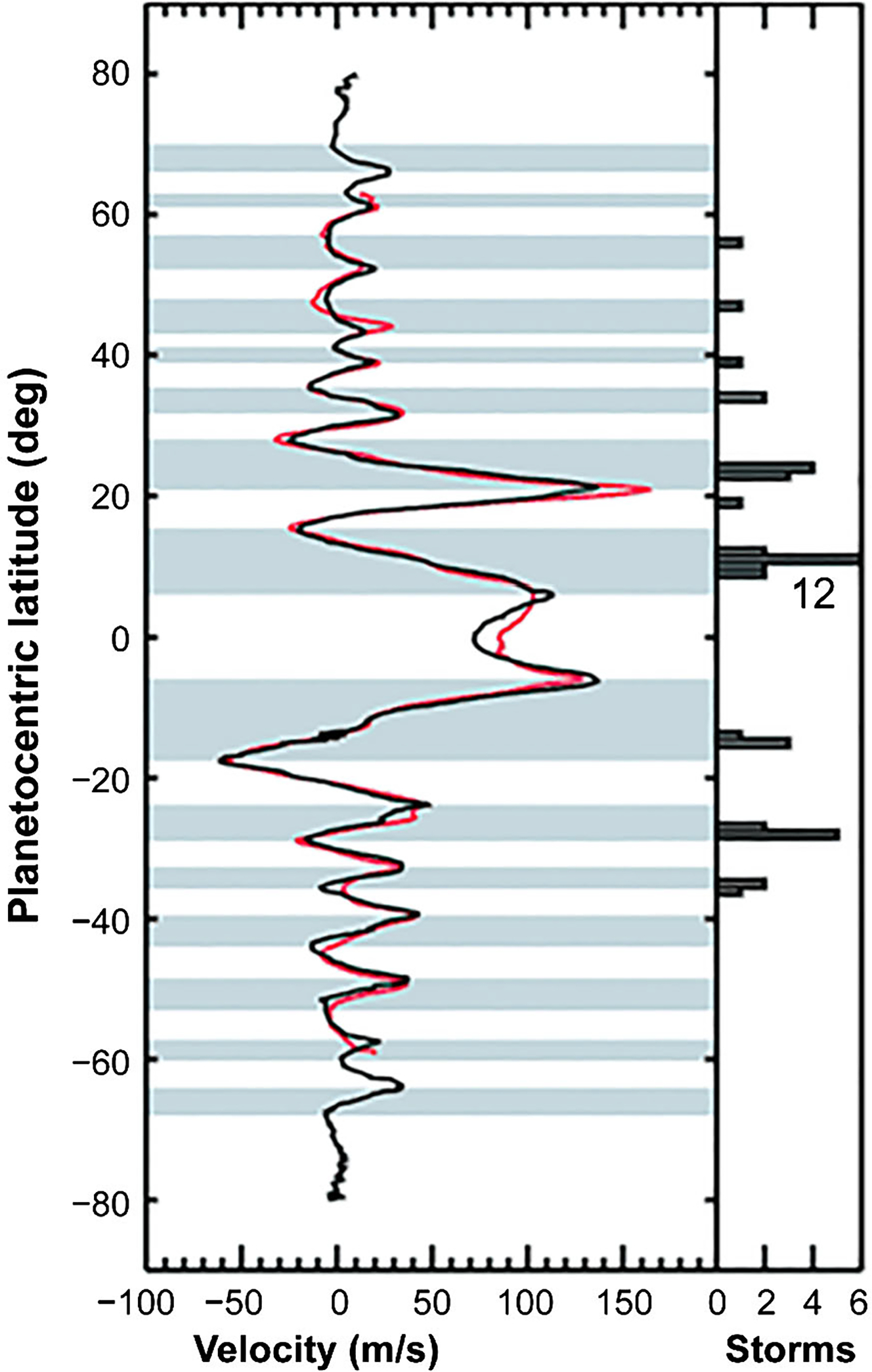
Atmospheric zonal velocity for Jupiter. The black curve is from Cassini ISS data in late 2000, and the red curve is from Voyager data in mid-1979. The jets are remarkably steady over this 21-year interval (Porco et al. 2003)

Jupiter’s zonal jets are remarkably steady in comparison with Earth’s jet streams, which change on time scales of 1 or 2 weeks (Fig. 19). Cassini ISS documented only one latitude outside the equator, near 21° planetocentric, where the jet speed had changed by a modest amount from Voyager in 1979 to Cassini in 2000 (Porco et al. 2003; Asay-Davis et al. 2011). Most of the observed variability is in the stratosphere at the equator (Garcia-Melendo et al. 2011a) and is part of a regular ~4 year oscillation similar to the quasi-biennial oscillation on Earth (Flasar et al. 2004; Simon-Miller et al. 2007; Simon-Miller and Gierasch 2010). Meridional transport is inferred indirectly from Cassini CIRS data that give the latitudinal distribution of C_2_H_2_ and C_2_H_6_, which have known chemical lifetimes and thereby track the age of air masses in the stratosphere (Liang et al. 2005; Zhang et al. 2013b). Vertical wind shear of the zonal winds is studied by tracking features at different wavelengths that probe different altitudes (Li et al. 2006c). Cassini data.

**Fig. 20:**
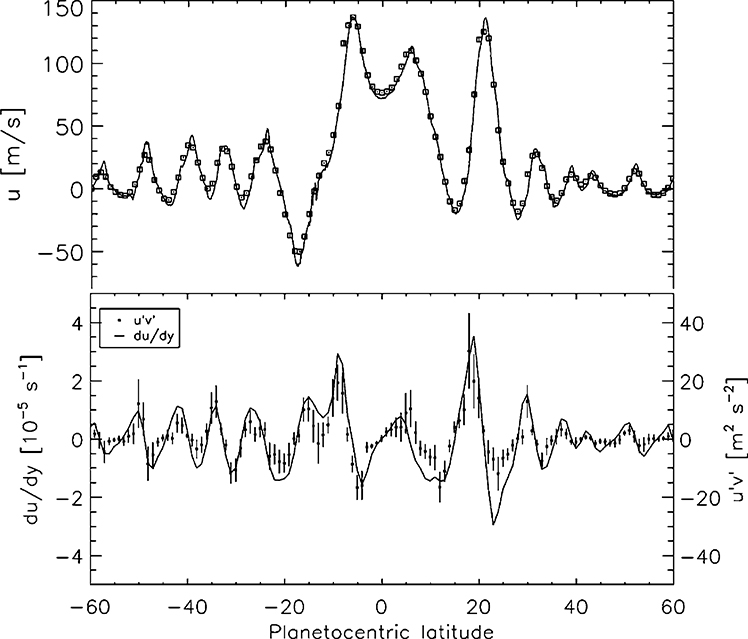
Eddy momentum transport for Jupiter from Cassini ISS. The eastward and northward eddy winds u′ and v′ are the departures from the zonal means. Their product $\bar{u^{\prime }v^{\prime }}$ averaged over longitude and multiplied by density is the northward eddy flux of eastward momentum. The fact that this quantity has the same sign as $\partial \bar{u}/\partial y$, which is the increase of the mean eastward wind with latitude, says that the eddies are putting energy into the jets and not the reverse (Salyk et al. 2006)

The alternating white and grey bands in Fig. 19 are the zones and belts, respectively. The zones are defined as having anticyclonic vorticity (clockwise in the northern hemisphere) and the belts as having cyclonic vorticity (counterclockwise in the northern hemisphere). The vertical strip on the right shows the latitudes where bright white storm clouds associated with lightning occurred (Dyudina et al. 2004), and that was usually in the belts. Lighting in the belts has been a puzzle since the Galileo days (Little et al. 1999; Gierasch et al. 2000) because except for the lightning storms, the belts are generally clear and the zones are cloudy. Thus clouds and chemical tracers imply upwelling in the zones (Gierasch et al. 1986), but lightning and the eddy momentum transport imply the opposite. Figure 20 shows that the eddy momentum transport is into the jets (Salyk et al. 2006). To balance the angular momentum, there must be a mean north-south flow that is converging in the zones and diverging in the belts (Ingersoll et al. 2000), implying upwelling in the belts, consistent with lightning. So there is conflicting evidence of upwelling and downwelling. The Cassini data shown in Figs. 19 and 20 have not solved this contradiction, but they have at least focused attention on it (Fletcher et al. 2020). The double Hadley cell suggested for Saturn in Fig. 2 may be the key for Jupiter as well.

Waves in a planetary atmosphere provide information about the medium in which they propagate and also about the sources that excite them. Mesoscale waves—wavelengths ~100 km—are visible from space if the crests and troughs are marked by clouds. The thunder following a lightning strike and the sonic boom following a meteor impact are examples of intense sound waves. Cassini could not detect sound waves, but it did detect thunderstorms and lightning (Dyudina et al. 2004; Porco et al. 2003; Baines et al. 2007). Voyager, Galileo, and New Horizons detected mesoscale wave trains with crests and troughs aligned north-south and wavelengths of ~300 km. Cassini apparently did not detect mesoscale waves (Arregi et al. 2009). They may have been absent at the time of the Cassini flyby or perhaps the waves were unobservable due to lack of cloud tracers. One theory says the mesoscale waves are propagating gravity waves (Flasar and Gierasch 1986), and another theory says they are shear instabilities (Bosak and Ingersoll 2002). The first theory requires an obstacle to the flow. The second theory requires strong shear and weak stratification. The measured phase velocities seem inconsistent with both models (Simon et al. 2015) given reasonable assumptions about the vertical wind shears, so the nature of the mesoscale waves is uncertain.

Cassini also detected large scale periodic patterns with wavelengths ranging up to 20,000 km. Circumpolar waves have wavelengths and phase speeds that suggest they are Rossby waves (Barrado-Izagirre et al. 2008, 2009). Simultaneous observations by ISS, UVIS, and CIRS allow one to probe the vertical structure and opacity sources of the waves (Li et al. 2006b). Radio observations allow one to study how the passage of a wave affects the distribution of ammonia (Cosentino et al. 2017). Cassini observations have been used to support various theories of wave-like features in the South Equatorial Belt—that they are either inertia-gravity waves or Rossby waves (Simon-Miller et al. 2012) or a pattern associated with baroclinic instability at that latitude (Rogers et al. 2016).

Wave mean-flow interactions and cascades of energy from one scale to another are fundamental processes in the dynamics of planetary atmospheres. One theory says that the zonal mean flow forms into a potential vorticity (PV) staircase. PV is a conserved dynamical tracer and the staircase consists of latitude bands with constant PV and sharp boundaries in between. However, quantitative analysis of the Cassini 70-day movie shows that the PV gradient reverses sign and is not zero as the staircase model predicts (Li et al. 2004; Read et al. 2006; Shetty and Marcus 2010). The 70-day movie was also used to show that the eddy momentum transport is into the jets from neighboring latitudes, meaning that the eddies are supplying energy to the jets and not the reverse (Fig. 20) (Salyk et al. 2006). The eddies could be getting their energy from convection (Li et al. 2006a) or the latitudinal gradient of radiative heating, or both (Schneider and Liu 2009). Fourier spectra of the 70-day movie data generally reveal an inverse energy cascade, where kinetic energy flows from intermediate scales to large scales (Choi and Showman 2011; Galperin et al. 2014; Hadjighasem and Haller 2016), but there is evidence of a forward cascade from intermediate scales down to small scales, which has been interpreted as energy input at the intermediate scales, perhaps associated with the radius of deformation through baroclinic instability (Young and Read 2017). Others have compared visible features in the 70-day movie with fine-scale features in their 3-dimensional numerical models to test the models’ treatment of unknown processes and parameters (Heimpel et al. 2005, 2016; Morales-Juberias and Dowling 2013).

### 8.2 Atmospheric Structure and Composition

**Fig. 21:**
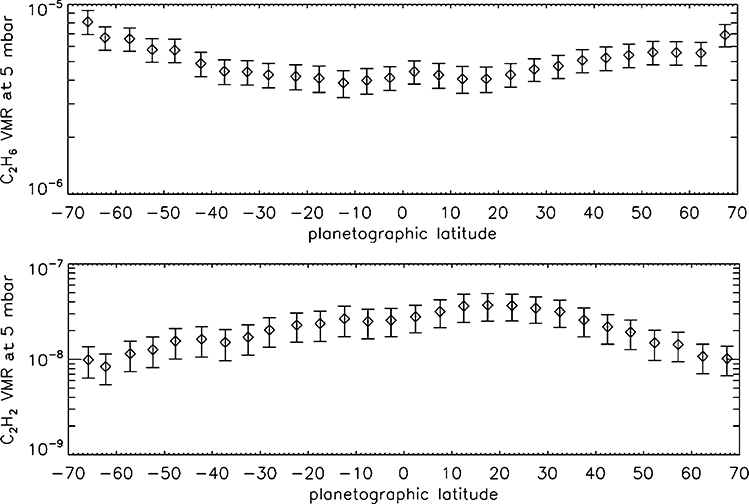
Chemical tracers in Jupiter’s atmosphere at 5 mbar from Cassini CIRS. Both acetylene and ethane are formed at low latitudes by solar UV. In photochemical equilibrium their abundances would be greatest at pressures less than 0.1 mbar. C_2_H_2_ has a short chemical time constant, ~ 3 × 10^7^ s, so its abundance reflects photochemical equilibrium with lower sunlight toward the poles. C_2_H_6_ has a long lifetime, ~ 3 × 10^10^ s, so its abundance at 5 mbar reflects transport by the meridional circulation. The time constant of this circulation is between the lifetimes of the two molecules (Nixon et al. 2007)

Reactive gases can serve as tracers that reveal regions of upwelling, downwelling, and meridional motion (Fig. 21). During the Jupiter flyby, Cassini CIRS measured the latitude distributions of C_2_H_2_ and C_2_H_6_, which are produced in the upper stratosphere by photodissociation of methane. The rate of production is greatest at the equator where the solar ultraviolet (UV) is greatest. C_2_H_2_ has a chemical time constant that is short compared to the meridional overturning time, so it decreases toward the poles in response to the reduced solar UV. C_2_H_6_ has a much longer chemical time constant than C_2_H_2_ – many hundreds vs. tens of years depending on altitude. One might expect it to have a flat distribution if it were carried poleward faster than the chemical reaction rate. But its abundance increases toward the poles (Nixon et al. 2007, 2010), and that is more difficult to explain (Hue et al. 2018). It is possible to fit the C_2_H_6_ distribution by adjusting parameters of the meridional circulation, but then the C_2_H_2_ distribution doesn’t fit. Independent estimates of the meridional circulation come from the distributions of HCN and CO_2_, which were measured by CIRS (Lellouch et al. 2006), but the problem remains.

Cassini CIRS also measured the latitude distributions of phosphine (Irwin et al. 2004; Fletcher et al. 2009a) and ammonia (Achterberg et al. 2006). The measurements refer to the upper troposphere, at the 100–500 mbar level, and they tell a consistent story. Both gases are removed from the atmosphere at upper levels, phosphine by photodissociation and ammonia by condensation, and both have maximum abundance at the equator and reduced abundance in the cyclonic belts on either side of the equator. This indicates rapid uplift or strong vertical mixing in the equatorial zone and descent in the neighboring belts. Probing still deeper into the troposphere, Cassini VIMS detected a 3 μm absorption that could be a mixture of NH_4_SH and NH_3_ (Brown et al. 2003; Sromovsky and Fry 2010) and provided new insights into the composition of fresh ammonia clouds (Sromovsky and Fry 2018).

Since the stratosphere is stably stratified, to maintain steady state the updrafts must be heated and the downdrafts must be cooled. The decrease of sunlight toward the poles is consistent with updrafts at the equator and downdrafts at the poles. CIRS measurements of stratospheric temperatures and abundances of radiatively active gases are used to model the radiative forcing (Zhang et al. 2013a, 2013b), which includes the effects of stratospheric aerosols (Zhang et al. 2013c, 2015).

Gaseous abundances can provide clues to planet formation. CIRS measured the nitrogen isotopic ratio in ammonia (Abbas et al. 2004; Fouchet et al. 2004a). The ratio is similar to that on the Sun but different from that on Earth, suggesting that the Earth was somehow isolated from the main reservoir of nitrogen during solar system formation. The hydrogen halides, on the other hand, are depleted relative to solar composition and support the halogens’ condensation in ammonium salts (Fouchet et al. 2004b).

The Cassini flyby provided insights into aurora-related phenomena on Jupiter as well. UVIS observations of the He 584 Å line were used to infer enhanced eddy mixing in the polar regions, which may account for the enhancement of heavy hydrocarbons there (Parkinson et al. 2006). Alternately, the enhancement of C_2_H_2_ and C_2_H_6_ in the polar regions could be explained by a combination of auroral-driven chemistry and advection by the meridional circulation (Sinclair et al. 2017, 2019). CIRS observations have also been used to study the sources of heating in the polar stratosphere from 1–10 mbar (Sinclair et al. 2018). The result is that heating by auroral soot particles dominates at 1 mbar altitude, and that heating by charged particles dominates at 10 μbar. Cassini VIMS observations have been used to produce the first maps of H_3_^+^ emission, temperature, and column density on Jupiter’s nightside (Stallard et al. 2015). The enhanced emission in the polar regions is produced by enhanced H_3_^+^ density rather than higher temperatures, which further points to the importance of auroral chemistry in that region.

Because of its high spatial and spectral resolution and full angular coverage during the flyby, Cassini obtained useful information about the clouds of Jupiter. Cloud color is a long-standing uncertainty: What chemical gives the Red Spot its redness? How many coloring agents are there? Comparison of laboratory experiments and visible spectra from Cassini VIMS suggests that the red chromophore is the product of photolyzed ammonia reacting with acetylene (Carlson et al. 2016; Baines et al. 2019). The chromophore occurs in an optically thin layer above the main cloud deck, and may require upward displacement of the air mass to form. A principal component analysis of Cassini ISS data indicates that the red chromophore explains most of the variance across the planet, but a second coloring agent is required to explain the brownish color of the cyclones in Jupiter’s North Equatorial Belt (Ordonez-Etxeberria et al. 2016; Teifel’ et al. 2018).

The vertical structure of the cloud layers is another long-standing unknown. Cassini CIRS thermal spectra were used to probe into the clouds at a wavelength of 7.18 μm, where the ammonia gas is relatively transparent (Matcheva et al. 2005). The result is that cloud base is at 0.9–1.1 bar, which is deeper than the nominal ammonia cloud base and might imply another, deeper cloud, possibly NH_4_SH. Cassini VIMS covers 4.5–5.1 μm, another wavelength range where the gas is relatively transparent. Holes in the cloud allow thermal radiation to space, so the holes appear as hot spots. The cloud is found to be spectrally flat and at a pressure of 1.2 bar or deeper (Giles et al. 2015). Pure NH_3_ cloud and pure NH_4_SH cloud do not match the spectra, and some kind of coating is implied. Despite the possibility of coatings, CIRS has detected the spectral signature of NH_3_ ice particles in the upper tropospheric clouds (Wong et al. 2004).

The planet’s global energy budget is a key to its evolution and internal processes, including the separation of helium from hydrogen in the metallic interior. To determine the energy budget accurately, one has to measure the thermal emission and scattered sunlight at all seasons, latitudes, longitudes, wavelengths, emission angles, incident angles, and phase angles. In practice, one samples these quantities and uses models to fill the gaps. Cassini CIRS, VIMS, and ISS all contribute to this estimate (Simon-Miller et al. 2006; Li et al. 2012; Sato et al. 2013; Mayorga et al. 2016). A comprehensive recent study (Li et al. 2018) reveals that Jupiter’s Bond albedo and internal heat, 0.503 ± 0.012 and 7.4785 ± 0.16 Wm^−2^ are both ~ 40% larger than the previous best estimates. The higher luminosity might come from greater helium differentiation, such that Saturn has formed a deep helium-rich shell or core (Mankovich and Fortney 2020).

## 9 Venus Atmosphere

The Venus flybys yielded useful data at a variety of wavelengths. The VIMS instrument detected thermal emission from the hot surface at wavelengths of 0.85 and 0.90 μm, and thereby provided a new technique for exploring the surface mineralogy (Baines et al. 2000). In an attempt to image the surface, the RADAR instrument was operated at 2.2 cm in both scatterometric and radiometric modes during the encounters, but only the thermal emission from the thick absorbing atmosphere was detected (Lorenz et al. 2001). The UVIS instrument studied the airglow of Venus, focusing on N_2_, C, O, and CO as clues to the excitation mechanisms and abundances (Hubert et al. 2010, 2012; Gerard et al. 2011). Lightning on Venus is a long-standing controversial subject. The RPWS instrument on Cassini searched for lightning signals in the frequency range 0.125 to 16 MHz and found none, even though they easily detected lightning at rates of 70 s^−1^ during the Earth flyby several months later (Gurnett et al. 2001).

## 10 Open Science Questions After Cassini

For an object as complex as Saturn, there will always be unanswered questions following a successful mission, and Cassini is no exception. Here is a list, by no means complete, of objectives that seem possible with future missions and expanded technologies.

### Water

Measure the global water abundance and determine its role in bringing heat to the surface. Determine the role of moist convection in maintaining the large-scale motions. A Saturn probe to the base of the water cloud is a challenging but possible objective. Juno-type microwave remote sensing, in addition, is possible and has the advantage of global coverage.

### Ammonia

Determine the global ammonia abundance, and map its distribution with depth over the planet. Currently available data are good down to only about 3 bars, which do not necessarily represent the deep well mixed abundance of ammonia. A Saturn probe to 10 bars could make this measurement, and microwave remote sensing is potentially valuable.

### Noble Gases

Determine Saturn’s noble gas abundances including helium, and their isotopic ratios, as done for Jupiter by the Galileo probe. A Saturn probe could measure noble gases to exquisite precision.

### Weather Layer

Determine the vertical profiles of temperature, winds, clouds, condensable gases, longwave and shortwave radiation, and turbulence levels. Again, a Saturn probe is needed. A cloud-resolving model like those used in tropical meteorology, but tailored to the giant planets, is both timely and valuable.

### Interior Motions

Determine Saturn’s rotation period if such a number exists, or determine the spread of periods if differential motions persist into the interior. Document the time dependence of motions in Saturn’s interior.

### Interior Structure

Using the power of ring seismology and gravity sounding, improve the understanding of Saturn’s internal equation of state, mass distribution, composition, and temperature distribution. Develop a fully coupled interior/weather-layer model as done for Earth’s ocean/atmosphere system.

## 11 Scientific Objectives

This section gives the Saturn portion of the scientific objectives for the prime mission and for the Cassini Solstice Mission, which was a mission extension. The former are listed either as S_AOn or J_AOn, with S for Saturn, J for Jupiter, AO for announcement of opportunity and n as a counter in the list. The latter are listed as SNnx and SCny, with S for Saturn, N for new phenomena to pursue, and C for change, both intrinsic and seasonally driven. Again nx and ny are counters. The list below is reproduced from Volume 1 of the Cassini Mission final report to NASA; Sect. 3, Science mission results; Saturn discipline science results. The full document is available at https://pds-rings.seti.org/cassini/.

- **Saturn Temperature, Clouds, Composition (S_AO1)**
  - **Temperature and clouds (S_AO1)** – Determine temperature field, cloud properties, and composition of the atmosphere of Saturn.
  - **Composition and chemistry (SN1c)** – Measure the spatial and temporal variability of trace gases and isotopes.
- **Saturn Winds and Weather (S_AO2)**
  - **Seasonal Variations (SC1a)** – Observe seasonal variations in temperature, clouds, and composition in three spatial dimensions.
  - **Saturn’s Winds (SC1b)** – Observe seasonal changes in the winds at all accessible altitudes coupled with simultaneous observations of clouds, temperatures, composition, and lightning.
  - **Storms (SN1b, SN2a)** – Observe the aftermath of the 2010–2011 storm. Study the life cycles of Saturn’s newly discovered atmospheric waves, south polar hurricane, and rediscovered north polar hexagon. Monitor the planet for new storms and respond with observations when they occur.
  - **Atmospheric dynamical processes (S_AO2)** – Measure the global wind field, including wave and eddy components; observe synoptic cloud features and processes.
  - **Saturn Lightning Sources and Morphology (S_AO6, SN2a)** – Investigate the sources and the morphology of Saturn lightning—Saturn Electrostatic Discharges (SED)—lightning whistlers.
- **Saturn Interior Structure and Rotation (S_AO3)**
  - **Saturn’s Rotation Rate (SN1a)** – Determine Saturn’s rotation rate and internal structure despite the planet’s unexpected high degree of axisymmetry.
  - **Saturn Formation and Evolution (S_AO5)** – Provide observational constraints (gas composition, isotope ratios, heat flux, ...) on scenarios for the formation and the evolution of Saturn.
- **Aurora, Chemistry, and Upper Atmosphere (SC2a)** – Observe the upper atmosphere and the aurora as it changes on all time scales—minutes to years—and is affected by seasonal and solar cycle forcing.
- **Jupiter atmospheric dynamics (J_AO1)** – Extend the time for studies of atmospheric dynamics beyond the period accessible to the Galileo nominal mission.
- **Jupiter Global Atmospheric Structure and Composition (J_AO2)** – Infer global atmospheric thermal structure and composition with instrumentation not carried by the Galileo Orbiter, complementing the local in situ measurements of the Galileo Probe.
- **Venus atmosphere observations** – No specific objectives were called out.
- **Cruise Planetary and Stellar Internal Oscillations (C_AO5)** – Attempt to detect internal oscillations of Saturn, Jupiter, and some stars.
- **Saturn Ionosphere-Magnetospheric Interaction (S_AO4)** – Study the diurnal variations and magnetic control of the ionosphere of Saturn.

## Data Availability

Cassini data can be accessed on the Planetary Data System (PDS) at https://pds.nasa.gov.

## Acronyms

AO: Announcement of Opportunity
CIRS: Composite Infrared Spectrometer
GCM: general circulation model
GRS: Great Red Spot
HST: Hubble Space Telescope
IR: infrared
ISS: Imaging Science Subsystem
MAPS: Magnetospheres and Plasma Science
PV: potential vorticity
QBO: quasi-biennial oscillation
RPWS: Radio and Plasma Wave Science
SAO: Semiannual Oscillation
SED: Saturn Electrostatic Discharges
SKR: Saturn kilometric radiation
UVIS: Ultraviolet Imaging Spectrograph
VIMS: Visual and Infrared Mapping Spectrometer
